# Effect of Ergothioneine on the Stability of Hyaluronic Acid-Based Wound-Healing Materials

**DOI:** 10.3390/polym18162033

**Published:** 2026-08-21

**Authors:** Tianyu Ma, Shuangshuang Qi, Junkai Liu, Dongjiao Li, Xia Li, Shiyue Hu, Fuhua Zheng, Ruiyan Wang, Yang Su, Yunjiao Chi, Xueqi Zhao, Zhen Qin, Hao Wu

**Affiliations:** 1 School of Pharmaceutical Engineering, Shenyang Pharmaceutical University, Shenyang 110016, China; matianyu001@stu.syphu.edu.cn (T.M.); qishuangshuang@stu.syphu.edu.cn (S.Q.); liujunkai@stu.syphu.edu.cn (J.L.); lidongjiao@stu.syphu.edu.cn (D.L.); chiyunjiao@stu.syphu.edu.cn (Y.C.); zhaoxueqi@stu.syphu.edu.cn (X.Z.); 2 Bloomage Biotechnology Co., Ltd., Jinan 250101, China; xiali@bloomagebiotech.com (X.L.); husy@bloomagebiotech.com (S.H.); zhengfh@bloomagebiotech.com (F.Z.); wangry@bloomagebiotech.com (R.W.); tenlions1@sina.com (Y.S.); 3 Key Laboratory of Regenerative Medicine Technology and Material Transformation of Hainan Province, Bloomage Biotechnology (Hainan) Co., Ltd., Haikou 570003, China

**Keywords:** hyaluronic acid, ergothioneine, hydrogel dressings, antioxidant excipient, stability, wound healing

## Abstract

Hyaluronic acid (HA)-based hydrogels are widely used as wound-healing materials and topical delivery systems because of their excellent biocompatibility, water retention capacity, and ability to promote cell migration. However, HA is prone to oxidative chain scission, which reduces molecular weight and compromises formulation stability and functional performance. This study evaluated the feasibility of ergothioneine (EGT) as a candidate antioxidant stabilizing excipient in a model HA-based wound-healing material. CCK-8 assays assessed the biocompatibility of EGT in L929 mouse fibroblasts after 24 h of exposure, and a stress-screening framework including Fenton oxidation, high-temperature/high-humidity treatment, light exposure, and quiescent storage at 4 °C was established. The results showed that Fenton oxidation markedly induced HA degradation, whereas EGT incorporation effectively protected HA structural integrity under oxidative stress. Cell scratch assays further demonstrated that EGT did not interfere with the ability of HA to promote cell migration. EGT may serve as a candidate antioxidant stabilizing excipient for HA-based wound-healing materials, improving HA structural and material stability under oxidative challenge while preserving HA-associated cell-migration function.

## 1. Introduction

Hyaluronic acid (HA) is a naturally linear glycosaminoglycan composed of repeating disaccharide units of D-glucuronic acid and N-acetyl-D-glucosamine. It is widely distributed in skin, synovial fluid, vitreous humor, and the extracellular matrix [1]. Because of its outstanding water retention capacity, biocompatibility, biodegradability, and ease of chemical modification, HA and its derivatives have found extensive applications in ophthalmic formulations, intra-articular injections, dermal fillers, tissue-engineering scaffolds, wound healing, and drug delivery systems [2,3,4]. In wound repair, HA not only maintains a moist local environment but also participates in the regulation of cell migration, angiogenesis, and extracellular-matrix remodeling, making it an important natural-polymer matrix for the construction of functional wound dressings [3,5,6].

Material stability is a critical factor for HA-based wound-healing materials and delivery systems, as it influences formulation development, storage quality, and functional maintenance. Wound sites, inflammatory tissues, and certain tumor microenvironments are typically associated with elevated levels of reactive oxygen/nitrogen species. Although moderate levels of reactive oxygen species (ROS) participate in antimicrobial defense and cellular signaling, excessive ROS can induce oxidative stress that damages cellular structures and interferes with inflammatory resolution, cell proliferation, angiogenesis, granulation-tissue formation, and extracellular-matrix reconstruction [6]. With respect to HA itself, reactive species such as hydroxyl radicals, peroxynitrite, and hypochlorite can trigger HA chain scission and depolymerization, leading to decreased molecular weight, altered rheological behavior, and attenuated material functionality [7,8,9]. Among these, the H_2_O_2_/Fe^2+^-mediated Fenton reaction generates highly reactive hydroxyl radicals and has been widely used to model free-radical-induced HA degradation under strong oxidative conditions [10,11,12]. Consequently, protecting HA chain integrity and preserving its material attributes under oxidative stress represent formulation-science challenges worthy of attention in the development of HA-based wound-healing materials and HA-based delivery systems.

From a broader pharmaceutical perspective, oxidative degradation is not restricted to polysaccharide materials but is also a common stability risk in complex formulations and biopharmaceutical products. Hydrogel-based delivery platforms have also been developed for the prolonged and controlled delivery of biomacromolecules such as insulin, highlighting the importance of polymer-network integrity, controlled-release performance, and biocompatibility in advanced delivery systems [13]. Therefore, the development of antioxidant excipients that combine good safety with formulation adaptability is of potential significance for the stabilization of oxidation-sensitive formulations.

Ergothioneine (EGT) is a natural sulfur-containing histidine derivative derived mainly from microorganisms and edible fungi, and it can accumulate in mammalian tissues via a specific transporter [14,15,16]. EGT scavenges multiple reactive oxygen and nitrogen species and can suppress free-radical generation by chelating transition metal ions [17,18,19]. EGT offers several advantages over other widely studied antioxidants for aqueous polysaccharide-based formulations. Ascorbic acid is a highly effective water-soluble antioxidant, but its chemical stability in formulations is readily compromised by oxygen, light, temperature, pH, and interactions with other formulation components, necessitating careful stabilization [20]. Glutathione (GSH) is a physiologically important thiol-containing antioxidant; however, its reduced form is readily oxidized to glutathione disulfide (GSSG), and its GSH/GSSG redox state is thus tightly coupled to the surrounding oxidative environment [21]. N-Acetylcysteine (NAC), another thiol-containing antioxidant in widespread clinical use, similarly raises formulation-specific stability concerns during storage [22]. In contrast, α-tocopherol is a lipid-soluble, chain-breaking antioxidant that acts primarily in lipid environments; its poor aqueous solubility can hinder homogeneous incorporation into highly aqueous formulations [23]. EGT scavenges a broad range of reactive species and is reported to be stable under light, heat, and acidic or alkaline conditions, although its interactions with transition-metal ions warrant consideration during formulation development [24].

In recent years, combinations of EGT and HA have begun to be explored in models of oxidative-stress-related injury, including radiation-induced skin injury and radiation enteritis, where EGT-HA systems have been reported to scavenge free radicals, reduce oxidative stress, and protect damaged tissues [25,26]. Nevertheless, existing studies have focused primarily on the biological protective effects of EGT or the overall therapeutic efficacy of composite materials, with less attention paid to the systematic evaluation of EGT as an antioxidant stabilizing excipient in terms of HA chain integrity, related material properties, and functional maintenance. Whether EGT can serve as a functional excipient that improves HA material stability under oxidative stress without compromising HA-associated repair functions remains to be elucidated.

Based on this background, this study used an HA wound-healing material as a model system to evaluate the feasibility of EGT as a candidate antioxidant stabilizing excipient. First, the biocompatibility of EGT toward L929 cells was assessed to provide a safety foundation for its incorporation into HA formulations. Next, HA and HA/EGT samples were subjected to Fenton oxidation, the corresponding non-oxidized control treatment, high-temperature/high-humidity treatment, light exposure, and quiescent storage at 4 °C. Molecular weight, molecular-weight distribution, appearance, and refractive index were then measured to compare the effects of different stress conditions on the stability of the HA/EGT system. After identifying a more discriminatory oxidative-stress model, the Fenton-oxidized and non-oxidized control groups were further characterized by macroscopic morphology of the lyophilized samples, scanning electron microscopy (SEM), Fourier Transform Infrared Spectroscopy (FTIR), rheological behavior, flowability, contact angle, and water retention capacity to clarify the protective effect of EGT on HA oxidative degradation and changes in material properties. Finally, cell scratch assays were employed to evaluate the influence of HA, EGT, and HA/EGT systems on L929 cell migration, thereby examining whether the pro-migratory function of HA was maintained after EGT addition. This study aimed to elucidate the stabilizing role of EGT in HA-based wound-healing materials from the perspectives of biocompatibility, structural stability, material properties, and functional maintenance, and to provide experimental support for its further application as a novel antioxidant excipient in HA-based delivery systems and other oxidation-sensitive formulations.

## 2. Materials and Methods

### 2.1. Materials

Sodium hyaluronate (molecular weight: 1.27 × 10^6^ Da, water content 8.1%) was purchased from Bloomage Biotechnology Corporation Limited (Jinan, China). Ergothioneine (EGT) was obtained from Dongying First Biochem Industrial Co., Ltd. (Dongying, China). Sodium dihydrogen phosphate (NaH_2_PO_4_) and disodium hydrogen phosphate (Na_2_HPO_4_) were purchased from Hunan Jiudian Hongyang Pharmaceutical Co., Ltd. (Changsha, China). Ferrous chloride (FeCl_2_) was obtained from Macklin Biochemical Co., Ltd. (Shanghai, China). Hydrogen peroxide (H_2_O_2_, 30% *w*/*w*) was purchased from Tianjin Fu Yu Fine Chemical Co., Ltd. (Tianjin, China). Ethylenediaminetetraacetic acid disodium salt (EDTA) was obtained from Tianjin Hengxing Chemical Reagent Manufacturing Co., Ltd. (Tianjin, China). Potassium bromide (KBr) was purchased from Tianjin Guangfu Technology Development Co., Ltd. (Tianjin, China). The CCK-8 assay kit was obtained from Beijing Solarbio Science & Technology Co., Ltd. (Beijing, China). RPMI-1640 medium, fetal bovine serum (FBS), penicillin–streptomycin solution, trypsin solution, and sterile PBS were purchased from Thermo Fisher Scientific Inc. (Waltham, MA, USA). L929 mouse fibroblast cells were provided by National Institutes for Food and Drug Control (Beijing, China).

### 2.2. EGT Biocompatibility Evaluation

Using ultrapure water as the solvent, a basic buffer system was prepared with NaH_2_PO_4_ (0.05 mg/mL) and Na_2_HPO_4_ (0.22 mg/mL). For the EGT biocompatibility evaluation, EGT was added to this buffer system to prepare sample solutions with prepared EGT concentrations of 0.5, 1, 2, 3, 5, 10, 15, and 20 mg/mL. These values refer to the concentrations of the prepared sample solutions before their addition to the cell culture system. The basic buffer system without EGT served as the control. The samples were thoroughly mixed and sterilized at 121 °C for 15 min using an autoclave (DSX-18L, Shanghai Shenan Medical Instrument Factory, Shanghai, China) before being used for cell experiments.

L929 mouse fibroblasts were selected as the model for cytotoxicity assessment in accordance with ISO 10993-5, which recommends established cell lines for the in vitro testing of medical devices [27,28]. As an immortalized, well-characterized cell line, L929 offers high reproducibility and avoids the donor-to-donor variability inherent in primary cells. Because cell type is a key variable influencing cytotoxicity outcomes [28], this standardized line enables consistent, comparable screening of excipient safety. Fibroblasts are key effector cells in wound healing and tissue repair, contributing to extracellular matrix deposition, wound contraction, and migration into the wound bed [29]; the L929 model is therefore biologically relevant to the wound-healing application addressed here. Given the preliminary scope of the present safety evaluation of EGT as a stabilizing excipient, a standardized screening cell line was considered appropriate at this stage.

Logarithmic-phase L929 cells were prepared as a single-cell suspension and seeded into 96-well plates at a density of 1.5 × 10^5^ cells/mL (100 µL per well). Cells were cultured at 37 °C in a humidified 5% CO_2_ incubator (BPN-CHH, Yiheng Scientific Instrument Co., Ltd., Shanghai, China) for 24 h to allow attachment.

After cell attachment, 10 µL of each prepared sample solution was added to each well, and the cells were cultured for another 24 h. The negative-control group consisted of cells treated with the EGT-free buffer sample. Sample-background wells contained only culture medium and the corresponding prepared EGT sample solution, whereas blank wells contained only culture medium. At the end of treatment, 10 µL of CCK-8 reagent was added to each well, and the plate was incubated in the dark for 1 h. Absorbance was then measured at 450 nm using a microplate reader (Varioskan LUX, Thermo Fisher Scientific, Waltham, MA, USA). Cell viability was calculated by Equation (1):

(1) $$\mathrm{Cell}\;\mathrm{viability}\left(\%\right)\;=\;\frac{A_{\mathrm{sample}}-A_{\mathrm{background}}}{A_{\mathrm{negative}\;\mathrm{control}}-A_{\mathrm{blank}}}\;\times \;100\%$$

where *A_sample_*, *A_background_*, *A_negative control_*, and *A_blank_* represent the absorbance of wells containing cells treated with EGT sample solutions, sample-background wells, negative-control wells, and blank wells, respectively (*n* = 8).

### 2.3. Stability Test Sample Preparation and Stress Treatments

An HA base formulation was prepared for stability evaluation using ultrapure water as the solvent. The formulation contained HA, NaH_2_PO_4_, and Na_2_HPO_4_ at concentrations of 15 mg/mL, 0.05 mg/mL, and 0.22 mg/mL, respectively. At 15 mg/mL, the HA concentration is well above the coil overlap concentration (≈1.4 mg/mL for 1 MDa HA [30]), so the high-molecular-weight HA chains form a physically entangled network without any chemical crosslinking agents.

After HA was fully swollen and a homogeneous system was formed, different concentrations of EGT were added to prepare HA/EGT samples with prepared EGT concentrations of 0.5, 1, 2, and 5 mg/mL. Samples without EGT served as the HA group. All samples were thoroughly mixed and then subjected to stability evaluation under stress conditions. Each group included three independent replicate samples.

To evaluate the stability of the HA/EGT system under different stress conditions, samples from each formulation group were assigned to five treatment conditions: Fenton oxidation, non-oxidized Fenton control, high-temperature/high-humidity treatment, light exposure, and quiescent storage at 4 °C in the dark. The quiescent storage group served as the unstressed storage control, whereas the non-oxidized Fenton control was included to match the EDTA addition and handling conditions of the Fenton oxidation group without exposure to FeCl_2_ or H_2_O_2_.

Hydroxyl radicals were generated by adding FeCl_2_ and H_2_O_2_ to the samples, achieving final concentrations of 1 mM Fe^2+^ and 20 mM H_2_O_2_. The reaction was carried out at 25 °C for 30 min. At the end of the 30 min reaction, EDTA was added to a final concentration of 10 mM to terminate the reaction. The corresponding non-oxidized control group did not receive FeCl_2_ or H_2_O_2_ but was supplemented with the same final concentration of EDTA. All other treatment conditions were identical.

High-temperature/high-humidity treatment was conducted at 40 ± 2 °C and 75 ± 5% relative humidity for 14 days. Light-exposure treatment was performed at room temperature for 1 h, with cumulative visible-light exposure not less than 1.2 × 10^6^ lx·h. After treatment, all samples were allowed to equilibrate to room temperature before subsequent characterization.

### 2.4. Measurement of Molecular Weight and Molecular-Weight Distribution

The molecular weight and molecular-weight distribution were determined by gel-permeation chromatography coupled with multi-angle light scattering and refractive-index detection (GPC-MALS-RI; Wyatt Technology Corporation, Santa Barbara, CA, USA). Ultrapure water was used as the mobile phase at a flow rate of 0.600 mL/min, and the refractive-index signal was employed for concentration detection. Data were processed with ASTRA software (version 8.0.2.5) using the Zimm model with a fit order of 1 and a dn/dc value of 0.1600 mL/g. The weight-average molecular weight (Mw), number-average molecular weight (Mn), peak molecular weight (Mp), and polydispersity index (PDI = Mw/Mn) were recorded to evaluate HA chain integrity and its changes under stress conditions.

### 2.5. Appearance Observation

After each treatment and equilibration to room temperature, the appearance of each sample was observed and recorded under identical background and lighting conditions, including color, transparency, homogeneity, and the presence of precipitation, phase separation, turbidity, or changes in gel state.

### 2.6. Measurement of Refractive Index

The refractive index of each sample was measured using an Abbe refractometer (WYA-2WAJ, Lichen Bangxi Instrument Technology Co., Ltd., Shanghai, China). Before measurement, samples were equilibrated at room temperature, and the instrument was calibrated according to the instructions provided by the manufacturer. Refractive index readings were recorded for each group.

### 2.7. Morphology of Lyophilized HA

Samples were first frozen at −80 °C for 12 h and then lyophilized under vacuum for 48 h using a freeze dryer (SCIENTZ-10ND, Ningbo Scientz Biotechnology Co., Ltd., Ningbo, China). The macroscopic morphology of the lyophilized samples was recorded by conventional digital imaging, including structural integrity, degree of looseness, and collapse status.

The lyophilized samples were then mounted on SEM sample holders and sputter-coated with gold before observation. Surface morphology was observed using a scanning electron microscope (SU8000, Hitachi, Tokyo, Japan) at an accelerating voltage of 10 kV. Representative SEM images were collected at selected magnifications to compare the morphological differences among the different groups.

### 2.8. FTIR

After freeze-drying, samples were mixed with dried KBr, pressed into pellets, and analyzed using an FTIR-650 spectrometer (Tianjin Port East Technology, Tianjin, China). The spectra were recorded at room temperature (25 °C) over the range of 400–4000 cm^−1^ at a resolution of 4 cm^−1^.

### 2.9. Rheological Characterization

Rheological characterization was performed using a Modular Compact Rheometer (MCR 302, Anton Paar GmbH, Graz, Austria) with a parallel-plate geometry (plate diameter: 35 mm; gap: 1.0 mm). An excess of sample was loaded and the extruded portion was trimmed after gap adjustment. All samples were equilibrated at 25 °C for 5 min after loading, and a solvent trap was used to minimize evaporation. Stress-sweep measurements were performed in oscillatory mode at a fixed angular frequency of 10 rad/s over a shear-stress range of 0.1–100 Pa. Steady-shear viscosity measurements were then performed over a shear-rate range of 0.1–100 s^−1^.

### 2.10. Flowability Evaluation

A 50 µL aliquot of each sample was deposited onto the surface of a horizontally placed clean glass slide and allowed to flow and spread under gravity and its own viscoelasticity. Timing began immediately after deposition, and spread images were recorded at 5 min. The spread area was measured with ImageJ (version 1.54g).

### 2.11. Contact-Angle Measurement

The static contact angle of each sample was measured at room temperature using a contact-angle measurement instrument (V5, Tianjin Yunfan Instrument Co., Ltd., Tianjin, China). A 10 µL aliquot of sample was dropped onto a glass slide (Yancheng Feizhou Glass & Plastic Co., Ltd., Yancheng, China), and the static contact angle after droplet spreading was recorded.

### 2.12. Water Retention Capacity Determination

Water retention capacity was evaluated because moisture retention is an important functional property of HA-based wound-healing materials. A 1 mL aliquot of each physically associated HA hydrogel sample was placed in a 6 mL Type I medium borosilicate glass vial (SCHOTT Pharmaceutical Packaging Co., Ltd., Lishui, China) and left open at 40 °C. Sample mass was recorded at 0, 1, 2, 4, 6, 8, 12, and 24 h. The mass-retention ratio was calculated according to Equation (2):

(2) $$\mathrm{Mass}\;\mathrm{retention}\left(\%\right)\;=\;\frac{W_{t}}{W_{0}}\times 100\%$$

where *W*_0_ is the initial sample mass and *W_t_* is the sample mass at the corresponding time point.

### 2.13. Cell Scratch Assay for Evaluating HA/EGT-Promoted Cell Migration

Cell scratch assay samples were prepared using the same HA base formulation as that used for the stability evaluation. EGT was added to prepare HA/EGT samples with prepared EGT concentrations of 0.5, 1, 2, 3, and 5 mg/mL. Samples without EGT served as the HA group. In addition, a culture-medium group and an EGT solution group prepared at 2 mg/mL were included. Before cell treatment, 200 µL of each prepared sample solution was first mixed with 2 mL of RPMI-1640 medium containing 1% FBS to prepare the sample-containing treatment medium. After thorough mixing, 2 mL of the resulting treatment medium was added to each well of a 6-well plate. Therefore, the prepared sample solutions were diluted 11-fold in the final treatment medium. All samples used for cell experiments were terminally sterilized at 121 °C for 15 min before use.

A cell scratch assay was performed with L929 mouse fibroblasts to evaluate the effect of the HA/EGT system on cell migration. Logarithmic-phase L929 cells were prepared as a single-cell suspension and seeded into 6-well plates at a density of 6 × 10^5^ cells/mL, with 2 mL of cell suspension added to each well. Cells were cultured until they reached approximately 90–100% confluence. A straight-line scratch was then made in the center of the cell monolayer with a sterile 200 µL pipette tip, detached cells were removed, and 2 mL of the sample-containing treatment medium was added [31]. This time point was designated 0 h. To minimize the contribution of cell proliferation to scratch-closure results, all groups were treated with RPMI-1640 medium containing 1% FBS. Cell-migration images were collected at 0, 3, 6, 12, and 24 h after scratching, and the scratch width at each time point was measured with ImageJ. The scratch-closure rate was calculated according to Equation (3):

(3) $$\mathrm{Scratc}h\text{-}\mathrm{closure}\;\mathrm{rate}\left(\%\right)\;=\frac{W_{0}-W_{t}}{W_{0}}\times 100\%$$

where *W*_0_ is the average scratch width at 0 h and *W_t_* is the average scratch width at the corresponding time point.

### 2.14. Statistical Analysis

All quantitative data are expressed as mean ± standard deviation (SD), and the number of replicate samples or replicate wells for each experiment is specified in the corresponding method description. Comparisons among multiple groups were performed using one-way analysis of variance (ANOVA). When two factors were involved, two-way ANOVA was used. Post hoc multiple comparisons were performed using Tukey’s or Dunnett’s test according to the experimental design. Significance levels are denoted as ns, not significant; * *p* < 0.05; ** *p* < 0.01; *** *p* < 0.001; and **** *p* < 0.0001. Data analysis and graphical representations were performed with GraphPad Prism version 10.1 (GraphPad Software, San Diego, CA, USA).

## 3. Results

### 3.1. Biocompatibility of EGT

**Figure 1:**
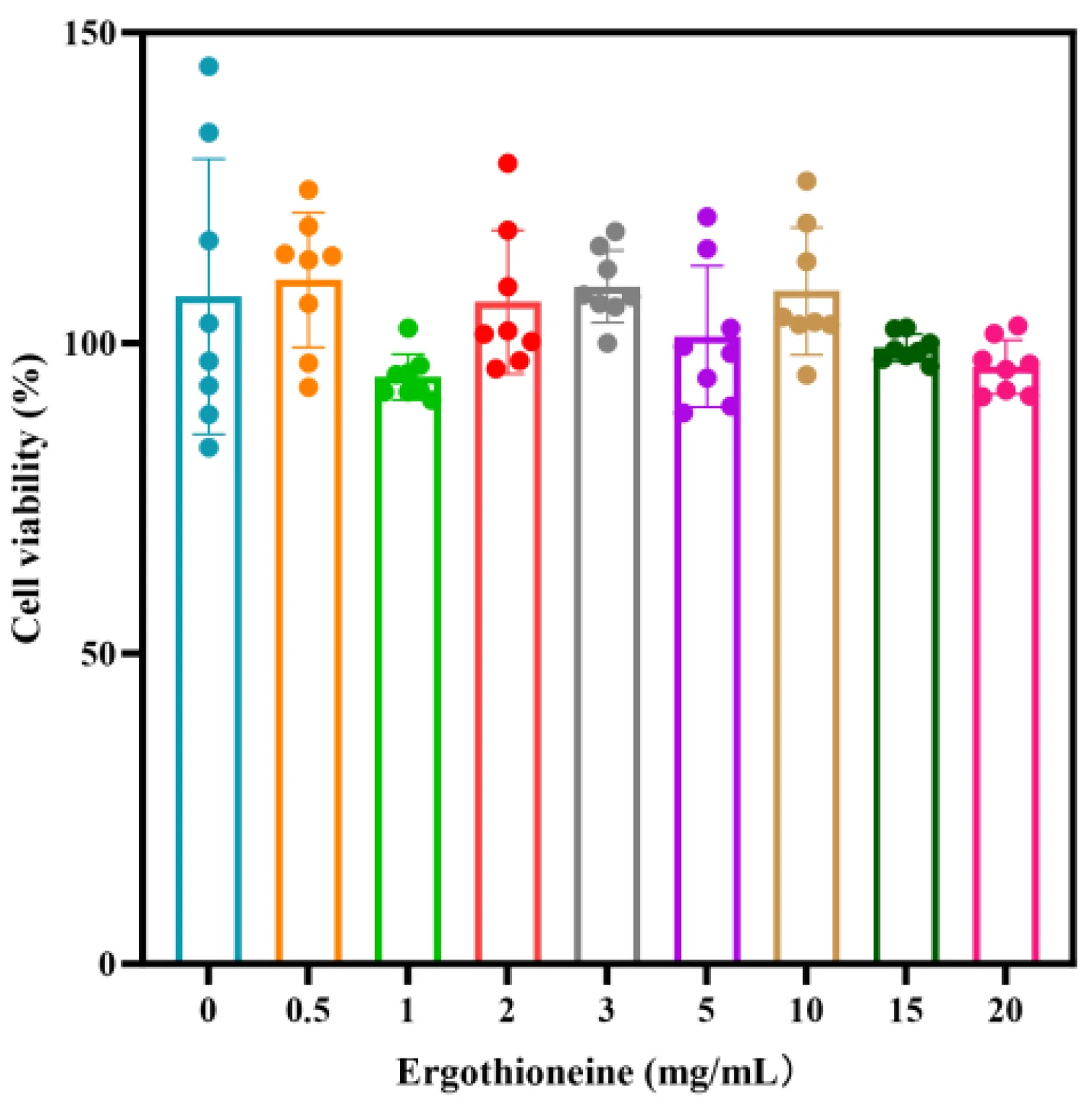
Cell viability of L929 cells after 24 h treatment with EGT at different prepared concentrations, as determined by the CCK-8 assay. Each point represents one replicate; bars and error bars indicate the mean and standard deviation, respectively (*n* = 8).

The biocompatibility of EGT sample solutions prepared at different concentrations toward L929 mouse fibroblasts was evaluated by the CCK-8 method. The results showed that, after the prepared EGT sample solutions were added to the cell system and incubated for 24 h, cell viability remained at a high level across the 0–20 mg/mL prepared-concentration range. Even for the EGT sample solutions prepared at 15 mg/mL and 20 mg/mL, cell survival rates were 99.24% and 96.15%, respectively. Some groups exhibited survival rates slightly above 100%, which was likely attributable to experimental variability in cell status, well-to-well differences, and the intrinsic fluctuation of the CCK-8 assay, rather than a genuine pro-proliferative effect (Figure 1). In summary, EGT did not exhibit apparent cytotoxicity within the prepared concentration range used in this study, providing a biocompatibility basis for its further evaluation as a functional excipient in HA formulations.

### 3.2. Molecular Weight and Molecular-Weight Distribution

**Figure 2:**
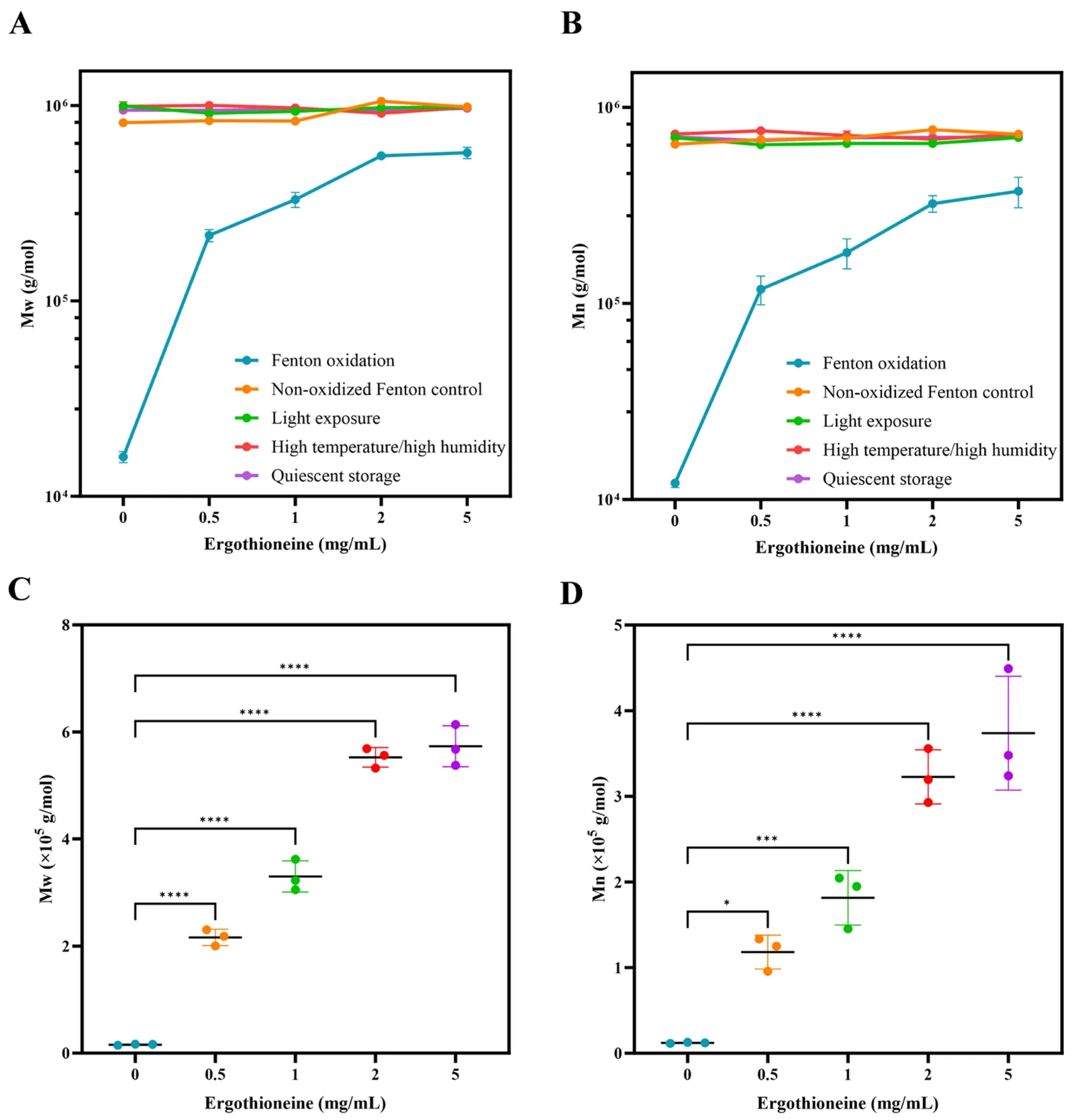
Effects of prepared EGT concentrations on HA molecular weight under different treatment conditions. (**A**) Mw under different treatment conditions. (**B**) Mn under different treatment conditions. (**C**) Mw of HA under Fenton oxidation. (**D**) Mn of HA under Fenton oxidation. Data are presented as mean ± SD (*n* = 3). Statistical comparisons in (**C**,**D**) were made versus the Fenton-oxidized HA group without EGT. * *p* < 0.05, *** *p* < 0.001, and **** *p* < 0.0001.

The molecular weight and molecular-weight distribution of HA samples after different stress treatments were determined by GPC-MALS-RI. The results showed that, under the non-oxidized control, light exposure, high-temperature/high-humidity treatment, and quiescent storage conditions, the Mn and Mw of all groups remained at broadly comparable levels without order-of-magnitude decreases, indicating that the HA backbone was generally stable under these non-oxidative or mild-stress conditions (Figure 2A,B; Table S1). By contrast, Fenton oxidation markedly reduced HA molecular weight. In the absence of EGT, the Mn and Mw of the Fenton-oxidized HA group decreased to 1.214 × 10^4^ g/mol and 1.592 × 10^4^ g/mol, respectively, indicating severe backbone scission under strong oxidative conditions.

Under Fenton oxidation, the extent of HA molecular-weight decline was progressively alleviated as the prepared EGT concentration increased. Compared with the HA group, the Mn values of the 0.5, 1, 2, and 5 mg/mL groups increased to 1.181 × 10^5^, 1.816 × 10^5^, 3.227 × 10^5^, and 3.737 × 10^5^ g/mol, respectively. The corresponding Mw values increased to 2.164 × 10^5^, 3.300 × 10^5^, 5.526 × 10^5^, and 5.731 × 10^5^ g/mol. Statistical analysis revealed that the Mw values of all EGT-treated groups were significantly higher than those of the HA group, and Mn exhibited the same trend (Figure 2C,D), demonstrating that EGT can effectively mitigate Fenton-oxidation-induced HA backbone scission.

To visualize the effect of EGT on HA oxidative degradation, representative GPC chromatograms of the non-oxidized HA control, the Fenton-oxidized HA group, and the groups with prepared EGT concentrations of 2 mg/mL and 5 mg/mL were compared (Figure S1). The main peak of the non-oxidized HA control was located in the shorter-retention-time region, indicating that the sample was dominated by higher-molecular-weight HA. The main peak of the Fenton-oxidized HA group shifted markedly toward longer retention times, indicating significant backbone degradation. After the addition of 2 mg/mL and 5 mg/mL EGT, the main peaks shifted forward relative to the Fenton-oxidized HA group, indicating an increase in higher-molecular-weight components and further supporting the protective effect of EGT against HA oxidative chain scission.

Polydispersity-index results showed that the PDI of the Fenton-oxidized HA group was 1.31. After the addition of EGT at prepared concentrations of 0.5 and 1 mg/mL, PDI increased to 1.86 and 1.84, respectively. When the prepared EGT concentration was raised to 2 and 5 mg/mL, PDI decreased to 1.72 and 1.55, respectively. These results suggest that, at low prepared EGT concentrations, the system may contain both oxidatively degraded low-molecular-weight segments and partially protected higher-molecular-weight segments, leading to a broadening of the molecular-weight distribution. As the prepared EGT concentration increased further, its inhibitory effect on backbone scission was enhanced, and the molecular-weight distribution gradually narrowed.

In summary, the molecular-weight-stabilizing effect of EGT on HA was manifested predominantly under oxidative-stress conditions. Under the present light-exposure, high-temperature/high-humidity treatment, and quiescent storage conditions, changes in HA molecular weight were modest, whereas Fenton oxidation rapidly induced HA backbone degradation, indicating that oxidative degradation is the more discriminatory destabilizing factor in this system.

### 3.3. Effects of Accelerated Stress Conditions on HA Morphology

**Figure 3:**
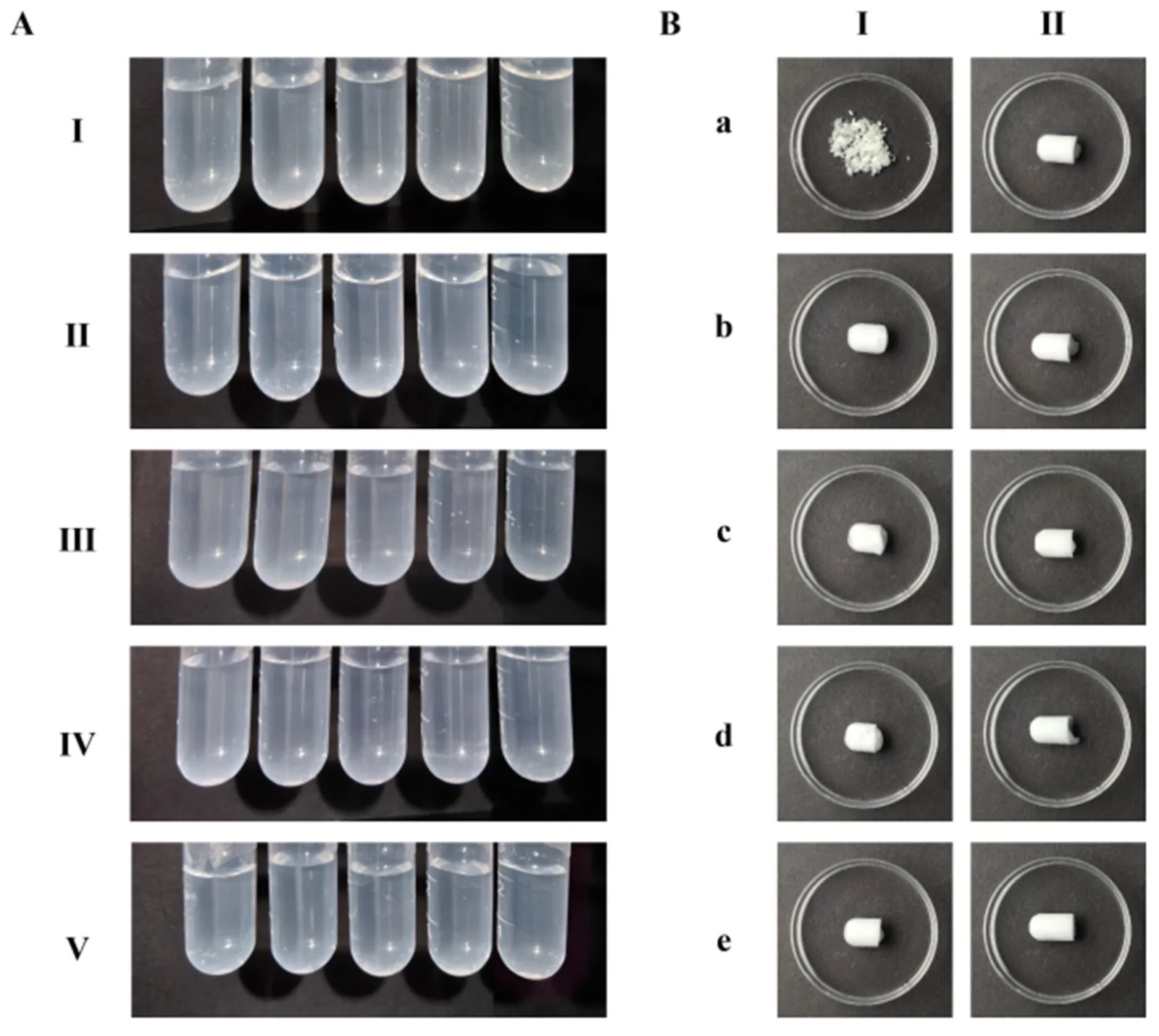
(**A**) Optical images of samples from each treatment group. (**I**) Fenton oxidation group. (**II**) Non-oxidized Fenton control group. (**III**) Light exposure group. (**IV**) High-temperature/high-humidity group. (**V**) Quiescent storage group. In each row, the prepared EGT concentrations from left to right are 0, 0.5, 1, 2, and 5 mg/mL. (**B**) Macroscopic morphology of lyophilized samples. (**I**) Fenton oxidation group. (**II**) Non-oxidized Fenton control group. The prepared EGT concentrations for (**a**–**e**) are 0, 0.5, 1, 2, and 5 mg/mL, respectively.

After quiescent storage, light exposure, high-temperature/high-humidity treatment, Fenton oxidation and the corresponding non-oxidized control treatment, the appearance of each group was observed. The results showed that all groups maintained relatively high transparency without obvious turbidity, phase separation, or precipitation, indicating that the HA hydrogel system possessed good macroscopic homogeneity (Figure 3A). Differences in appearance among samples with different prepared EGT concentrations under the same stress condition were minor, suggesting that EGT addition did not impair the clarity or appearance stability of the system.

Lyophilized cake morphology results showed that the Fenton-oxidized sample without EGT addition was markedly loose and had poor integrity, whereas EGT-supplemented samples mostly exhibited relatively intact block-like structures (Figure 3B).

**Figure 4:**
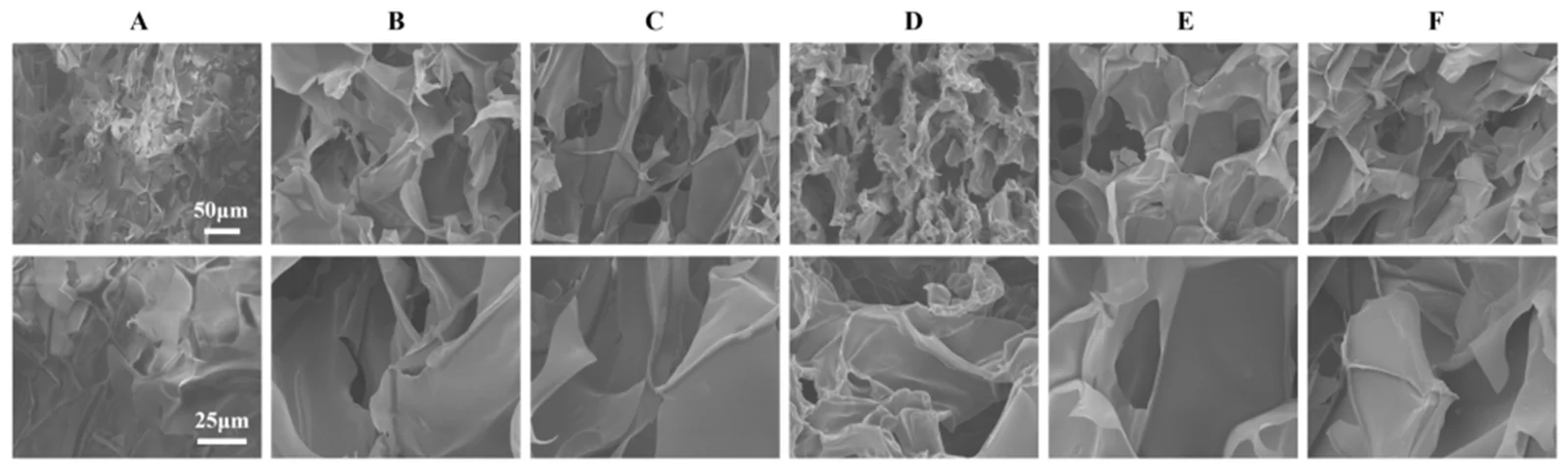
SEM micrographs of Fenton-oxidized samples, with the non-oxidized Fenton control included for comparison. (**A**) Fenton-oxidized HA without EGT. (**B**–**E**) Fenton-oxidized HA samples with prepared EGT concentrations of 0.5, 1, 2, and 5 mg/mL, respectively. (**F**) Non-oxidized Fenton control without EGT. The upper and lower rows show representative images at different magnifications. Scale bars are 50 µm and 25 µm, respectively.

SEM observation further revealed the microstructural changes in the samples after Fenton oxidation (Figure 4). The Fenton-oxidized HA sample without EGT showed a relatively loose and fragmented lamellar structure, suggesting oxidative damage to the HA gel network. In contrast, EGT-supplemented samples exhibited better-preserved sheet-like and porous microstructures, with less obvious structural disruption, and their morphology was closer to that of the non-oxidized Fenton control. These observations further support that EGT can partially preserve the microstructural integrity of the HA network under oxidative stress.

### 3.4. Effect of Fenton Oxidation on the Refractive Index of the Samples

**Figure 5:**
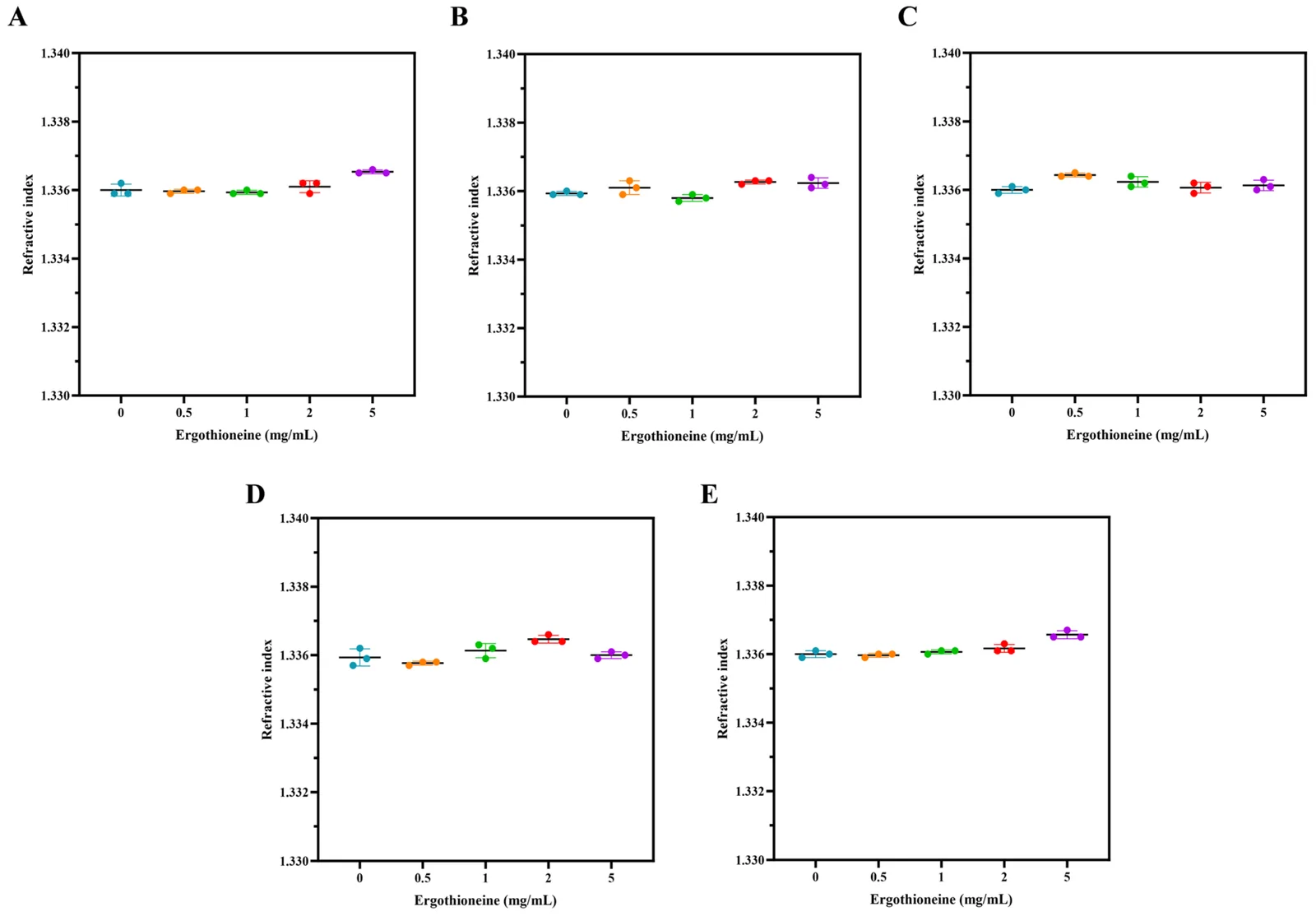
Refractive index of HA formulations with different prepared EGT concentrations under different treatment conditions. (**A**) Fenton oxidation group; (**B**) non-oxidized Fenton control group; (**C**) light-exposure group; (**D**) high-temperature/high-humidity group; (**E**) quiescent storage group. Data are presented as mean ± SD (*n* = 3).

Under different treatment conditions and prepared EGT concentrations, the refractive indices of all samples fluctuated within a narrow range, with all measured mean values concentrated between 1.3358 and 1.3366, and no consistent dose-dependent trend was observed. These results indicate that no substantial changes occurred in the overall composition or optical properties of the system (Figure 5). Compared with the GPC results, refractive index showed lower sensitivity to HA backbone oxidative scission and is therefore more suitable as an auxiliary indicator of overall appearance and optical stability.

### 3.5. FTIR Analysis

**Figure 6:**
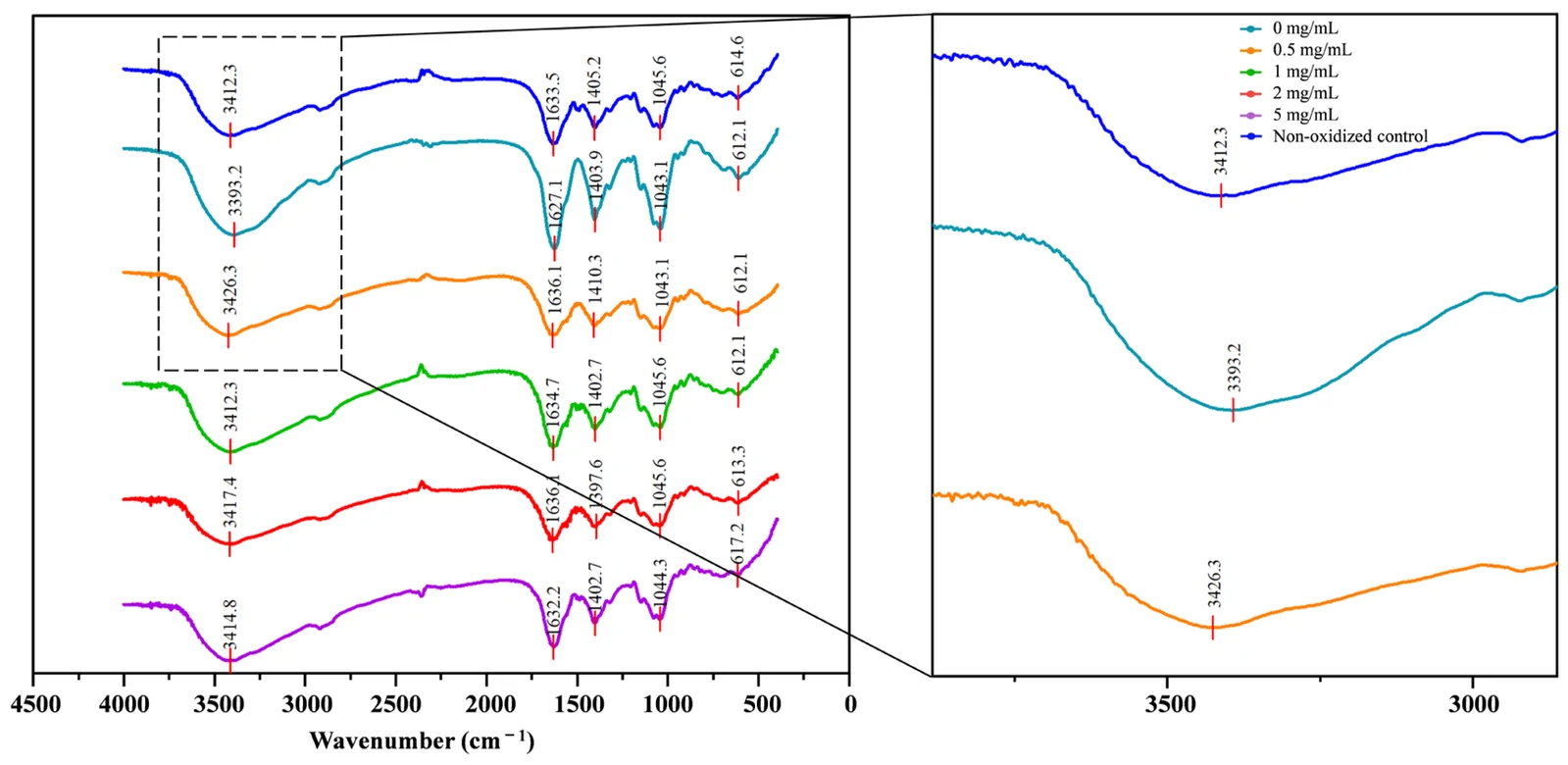
FTIR results of the Fenton oxidation group and its control group. Full infrared spectra with peak-position distribution and a magnified view of the 3000–3500 cm^−1^ region.

FTIR results showed that both Fenton-oxidized samples and non-oxidized controls displayed characteristic infrared absorption peaks typical of HA (Figure 6), including a broad O-H/N-H stretching vibration peak at approximately 3400 cm^−1^, a C-H stretching vibration peak at approximately 2920 cm^−1^, a carbonyl/amide-related absorption peak at approximately 1630 cm^−1^, a carboxyl-related vibration peak at approximately 1405 cm^−1^, and sugar-ring skeletal vibrations in the 1150–1040 cm^−1^ region corresponding to C-O and C-O-C bonds. No obvious new characteristic absorption peaks were observed among the different treatment groups, indicating that neither Fenton oxidation nor EGT addition induced the formation of new covalent bonds or skeletal structural changes in the HA backbone that could be clearly identified by FTIR.

Further comparison revealed that the HA sample without EGT, after Fenton oxidation, exhibited certain peak-shift and peak-shape changes in the broad peak near 3400 cm^−1^, the absorption peak near 1630 cm^−1^, and the fingerprint region, suggesting that oxidative stress may affect the hydrogen-bonding environment surrounding HA molecules, the state of bound water, and local structure. As the prepared EGT concentration increased, the above peak-position and peak-shape changes were generally attenuated, and the differences among the groups were relatively reduced, indicating that EGT can diminish the perturbation of Fenton oxidation on HA intermolecular interactions and the local microenvironment, thereby contributing to the structural stability of the HA system.

Overall, the FTIR results suggest that the protective effect of EGT against HA oxidative damage does not originate from the formation of new covalent cross-linking structures but is more likely related to its ability to attenuate oxidation-induced changes in the hydrogen-bond network, bound-water state, and local molecular environment.

### 3.6. Rheological Behavior Under Fenton Oxidation

**Figure 7:**
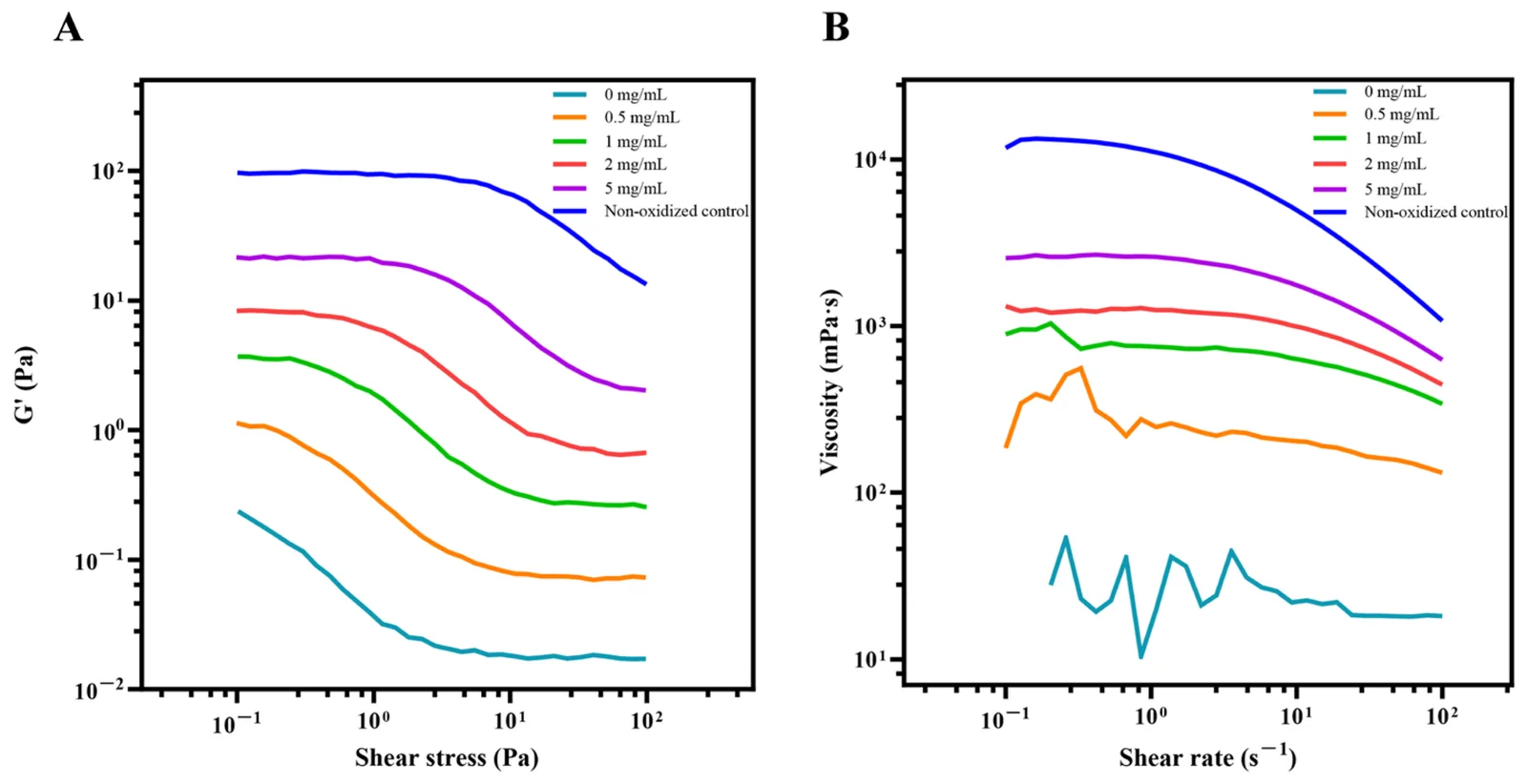
Rheological behavior of HA/EGT samples under Fenton oxidation. (**A**) Storage modulus (G′) as a function of shear stress in Fenton-oxidized HA samples containing different prepared EGT concentrations and in the non-oxidized control. (**B**) Apparent viscosity as a function of shear rate.

Rheological measurements were performed to further evaluate the effect of EGT on the viscoelastic and flow properties of HA under Fenton oxidation. In the stress-sweep test, the storage modulus (G′) of the Fenton-oxidized HA group without EGT was markedly lower than that of the non-oxidized control over the tested shear-stress range, indicating severe weakening of the HA network after oxidative degradation (Figure 7A). In the initial low-stress region, defined as the first three data points of the stress sweep at approximately 0.10–0.16 Pa, the average G′ values of the 0, 0.5, 1, 2, and 5 mg/mL EGT groups increased from approximately 0.21 to 21.48 Pa, whereas that of the non-oxidized control was approximately 95.91 Pa. This concentration-dependent increase in the initial G′ value suggests that EGT partially preserved the elastic response of HA under oxidative stress.

The viscosity–shear rate curves further showed that the non-oxidized control exhibited the highest apparent viscosity and pronounced shear-thinning behavior, which is characteristic of a non-Newtonian pseudoplastic polymer system (Figure 7B). Fenton oxidation markedly reduced the viscosity of HA, and the 0 mg/mL EGT group showed a low-viscosity profile with a much weaker shear-thinning tendency, suggesting extensive HA chain scission and loss of polymer-chain entanglement. With increasing prepared EGT concentration, the apparent viscosity of the Fenton-oxidized samples progressively increased across the tested shear-rate range. For example, at 1.08 s^−1^, the apparent viscosity increased from 19.718 mPa·s in the 0 mg/mL EGT group to 2599.6 mPa·s in the 5 mg/mL EGT group, although it remained lower than that of the non-oxidized control.

Overall, the stress-sweep and viscosity-curve results demonstrate that Fenton oxidation substantially impaired the rheological properties of HA, whereas EGT concentration-dependently mitigated the oxidation-induced loss of storage modulus and apparent viscosity. These findings are consistent with the molecular-weight results and further support the protective effect of EGT against Fenton-oxidation-induced HA chain scission.

### 3.7. Effect of Fenton Oxidation on Flowability of the Samples

**Figure 8:**
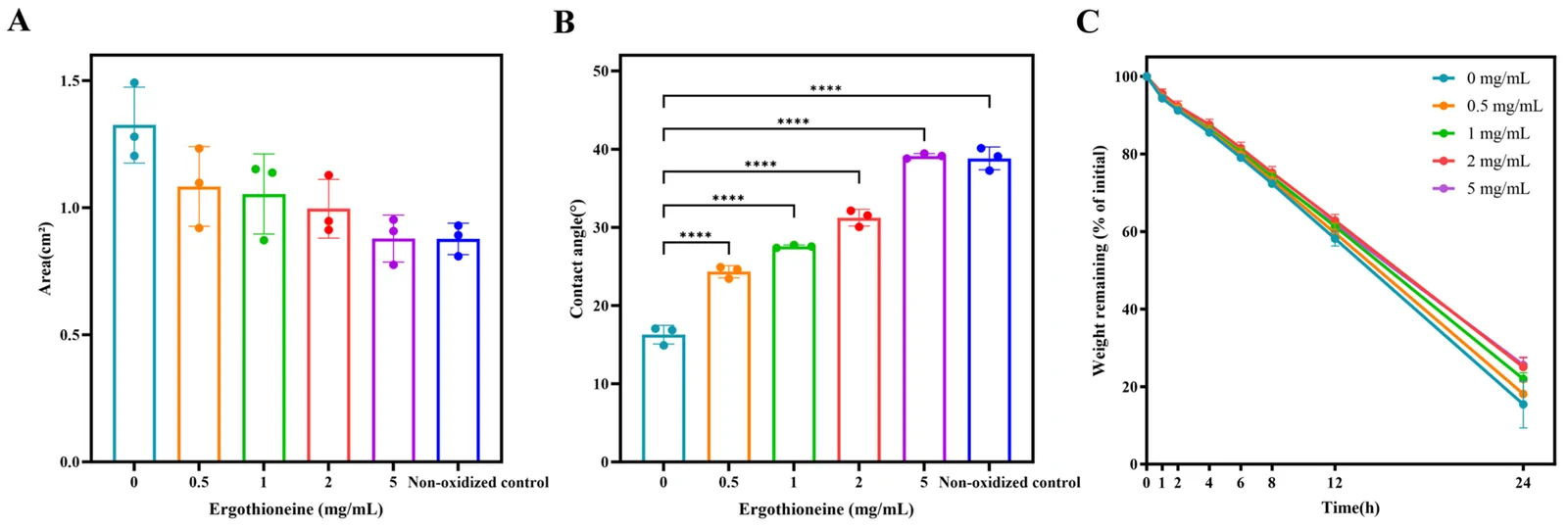
Flowability, contact angle, and water retention capacity of HA formulations after Fenton oxidation. (**A**) Flowability of Fenton-oxidized samples, with the non-oxidized Fenton control included for comparison. (**B**) Contact angle of Fenton-oxidized samples, with the non-oxidized Fenton control included for comparison. Statistical comparisons were made versus the Fenton-oxidized HA group without EGT. (**C**) Mass retention ratios of Fenton-oxidized samples at different time points. For (**A**,**B**), individual points represent independent replicates, and bars and error bars indicate the mean and SD, respectively (*n* = 3). For (**C**), the 0 h mass of each sample was used as the initial value to calculate the mass retention ratio at each time point; data are presented as mean ± SD (*n* = 3). **** *p* < 0.0001.

The flowability test showed that the spread areas of Fenton-oxidized samples with different prepared EGT concentrations differed markedly (Figure 8A). The HA group exhibited the largest spread area (1.325 ± 0.149 cm^2^). As the prepared EGT concentration increased from 0.5, 1, and 2 mg/mL to 5 mg/mL, the sample spread areas decreased to 1.084 ± 0.157, 1.054 ± 0.158, 0.996 ± 0.116, and 0.879 ± 0.093 cm^2^, respectively. The spread area of the non-oxidized control group was 0.877 ± 0.062 cm^2^.

A larger spread area on the glass slide indicates greater fluidity and lower viscosity of the system. The HA group displayed the largest spread area after Fenton oxidation, indicating poor structural stability of the HA system under oxidative stress. After EGT addition, the sample spread area decreased progressively with increasing prepared EGT concentration, indicating that the HA system at higher prepared EGT concentrations showed a lower degree of flow/spreading and better structural retention. These results demonstrate that EGT contributes to maintaining the structural stability of the HA gel network under Fenton oxidation.

### 3.8. Effect of Fenton Oxidation on the Contact Angle of the Samples

Contact-angle measurement results showed that Fenton oxidation stress markedly altered the surface wetting behavior of the samples. Under Fenton oxidation, the average contact angle of the HA group was 16.27°, whereas the average contact angles of the 0.5, 1, 2, and 5 mg/mL groups were 24.33°, 27.56°, 31.24°, and 39.12°, respectively. The non-oxidized control group was 38.82°. The HA group exhibited the lowest contact angle, indicating enhanced spreading and significantly altered interfacial properties after oxidative degradation (Figure 8B). These results indicate that EGT can alleviate the effect of Fenton oxidation on the interfacial properties of the HA system. The standard deviation across all groups ranged from 0.19° to 1.46°, reflecting good reproducibility of the contact-angle measurements. All samples maintained high hydrophilicity.

### 3.9. Effect of Fenton Oxidation on Water Retention Capacity of the Samples

Under Fenton oxidation, the mass-retention ratio of all groups decreased continuously over time, indicating significant water loss during open storage at 40 °C (Figure 8C). During the 0–8 h interval, the curves of the different prepared EGT concentration groups were relatively close, and intergroup differences were modest. After 12 h, curve separation gradually increased, indicating that the effect of EGT on water-retention capacity became more pronounced at the middle and later time points. At 12 h, the mass-retention ratio of the HA group was approximately 58.2%, whereas the 1, 2, and 5 mg/mL groups were approximately 61.3%, 62.9%, and 61.9%, respectively. By 24 h, the mass-retention ratios of the HA, 0.5, 1, 2, and 5 mg/mL groups were approximately 15.4%, 18.1%, 22.0%, 25.0%, and 25.6%, respectively. Compared with the HA group, all EGT-supplemented groups showed improved mass-retention ratios at 24 h, with the 2 and 5 mg/mL groups exhibiting the best water-retention performance.

From a concentration-effect perspective, the improvement in mass-retention ratio by EGT generally increased with the prepared EGT concentration but gradually plateaued at higher concentrations. This result is consistent with the molecular-weight and viscosity data, indicating that EGT helps delay water loss from the system by protecting HA chain integrity and gel-network integrity.

### 3.10. Effect of the HA/EGT System on L929 Cell Migration

**Figure 9:**
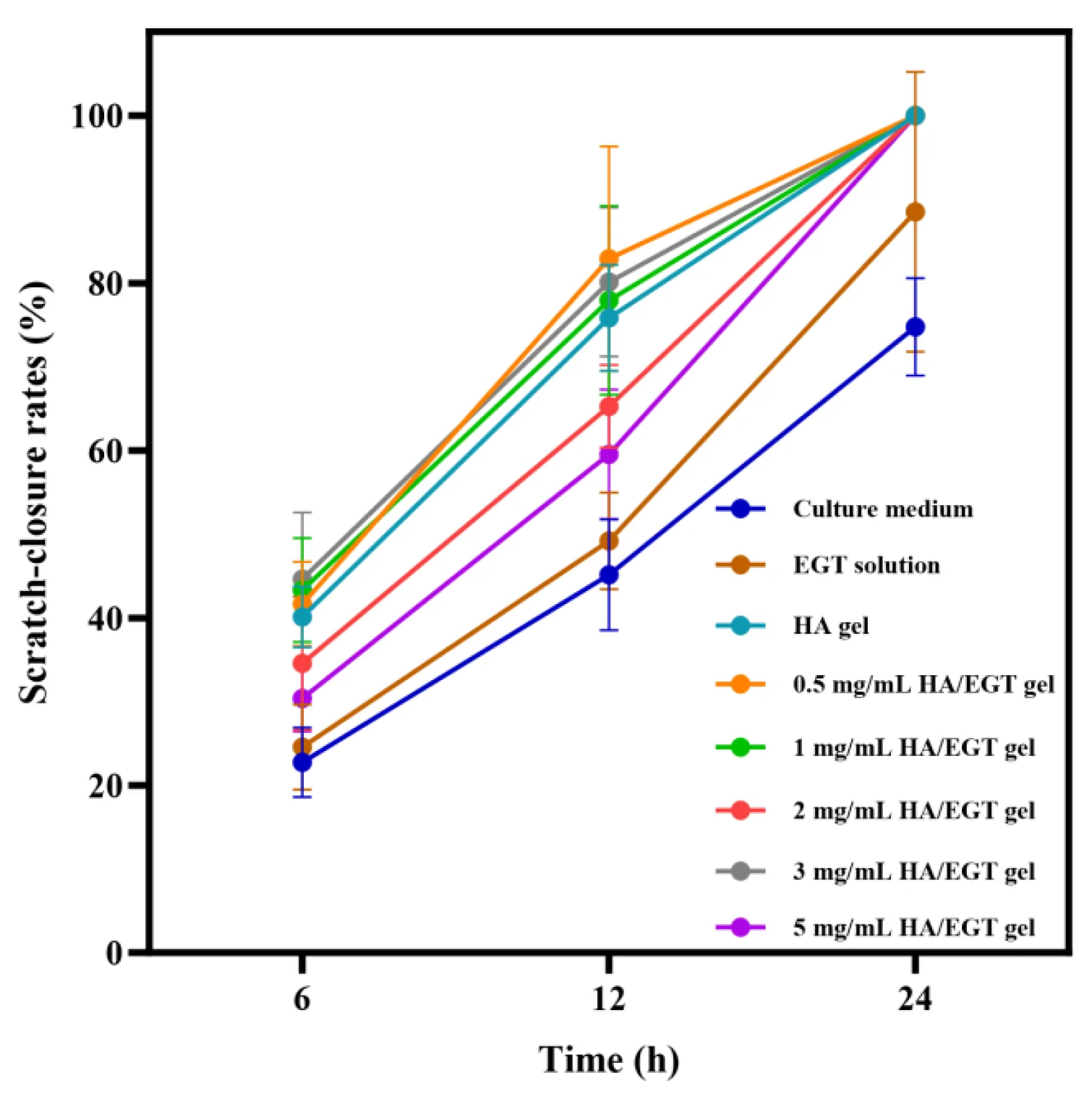
Quantitative analysis of scratch-closure rates of L929 cells treated with culture medium, EGT solution, HA hydrogel, and HA/EGT hydrogel groups with different prepared EGT concentrations. Quantitative scratch-closure rates at 6, 12, and 24 h are shown. Data are presented as mean ± SD (*n* = 3).

**Figure 10:**
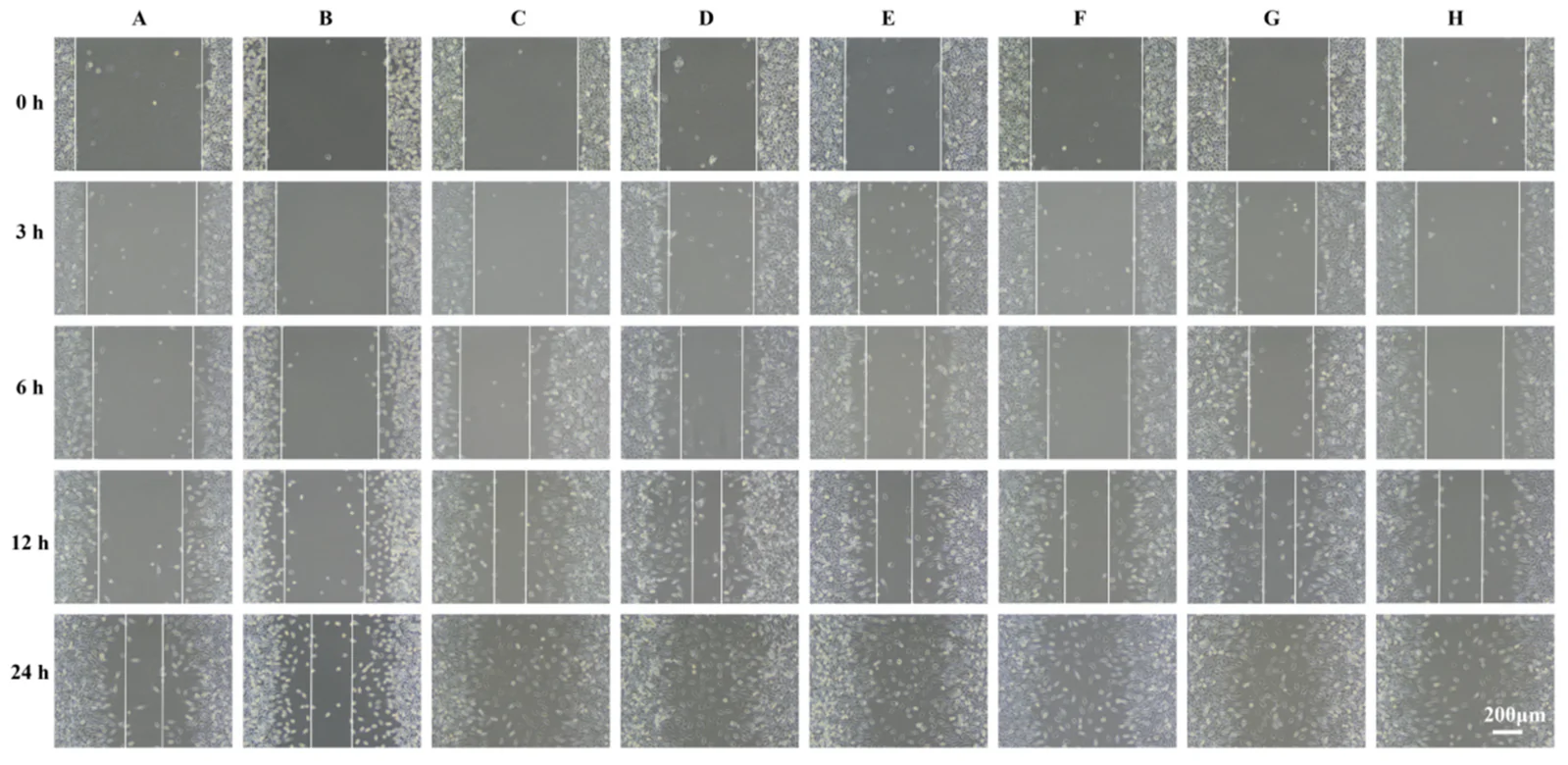
Representative microscopic images of the cell scratch assay at 0, 3, 6, 12, and 24 h. (**A**) Culture medium group. (**B**) EGT solution group. The EGT solution was prepared at 2 mg/mL. (**C**) HA hydrogel group. (**D**–**H**) HA/EGT hydrogel groups with prepared EGT concentrations of 0.5, 1, 2, 3, and 5 mg/mL, respectively. Scale bar = 200 µm.

At 6 h, the scratch-closure rates of the culture-medium group and the EGT solution group prepared at 2 mg/mL were 22.76% ± 4.16% and 24.58% ± 5.07%, respectively, with the two groups at broadly comparable levels (Figure 9; representative images are shown in Figure 10). By contrast, the overall closure rates of the hydrogel groups at 6 h were higher: the HA group and the HA/EGT groups with prepared EGT concentrations of 0.5, 1, 2, 3, and 5 mg/mL were 40.10% ± 3.67%, 41.66% ± 5.01%, 43.35% ± 6.23%, 34.60% ± 7.99%, 44.64% ± 7.95%, and 30.37% ± 3.96%, respectively. These results indicate that, in the early stage after scratching, the HA hydrogel system was generally more conducive to cell migration toward the scratched region, whereas the EGT solution did not exhibit a pro-migratory effect.

The 6 h closure values did not follow a monotonic concentration-dependent trend: the 0.5, 1, and 3 mg/mL groups showed closure rates comparable to or higher than that of the HA group, whereas the 2 and 5 mg/mL groups were slightly lower. In the absence of a clear dose–response relationship, this non-monotonic fluctuation most likely reflects experimental variation, as the manual scratch assay is inherently subject to variability in the initial scratch width [32]. Consistent with this interpretation, all HA-containing groups achieved complete (100%) scratch closure at 24 h, confirming that EGT addition did not compromise the pro-migratory function of HA.

At 24 h, the closure rate of the culture-medium group was 74.76% ± 5.82%, and that of the EGT solution group prepared at 2 mg/mL was 88.52% ± 16.72%. Although the mean value of the EGT solution group was higher than that of the culture-medium group, intragroup variability was substantial. The hydrogel groups overall still exhibited high closure rates. The HA group and the HA/EGT groups with prepared EGT concentrations of 0.5, 1, 2, 3, and 5 mg/mL all reached 100.00%, indicating that scratch closure was essentially achieved by the observation endpoint.

Overall, the culture-medium group and the EGT solution group displayed weaker migration performance than the hydrogel groups. The EGT solution group was comparable to the culture-medium group in the early and middle stages and did not show a clear independent pro-migratory effect. On the other hand, both the HA/EGT and HA groups maintained high closure rates, indicating that EGT addition did not impede the ability of HA to promote cell migration and demonstrating good compatibility between EGT and HA.

## 4. Discussion

Pathological wound microenvironments are often accompanied by elevated ROS levels, which can affect cellular behavior and may also damage oxidation-sensitive polymeric materials used for local delivery and tissue repair. Recent studies on ROS-scavenging nanomaterials and antioxidant hydrogel dressings further support the potential of biomaterial-based strategies for restoring redox balance in wound microenvironments [33,34]. In this context, the present study evaluated EGT as a candidate antioxidant stabilizing excipient for HA-based wound-healing materials. The biocompatibility results showed that EGT did not cause an apparent decrease in L929 cell viability within the prepared concentration range used in this study, providing a basic safety foundation for its incorporation into HA formulations. This is consistent with previous reports on the good safety profile and biological compatibility of EGT [35,36].

The strong destabilizing effect of Fenton oxidation can be explained by the radical-induced depolymerization mechanism of HA. In the Fe^2+^/H_2_O_2_ system, hydroxyl radicals are generated and can attack HA mainly through hydrogen abstraction from C–H sites on the sugar residues of the polysaccharide backbone. The resulting carbon-centered radicals may react with oxygen to form peroxyl radical intermediates, followed by rearrangement, β-scission, and cleavage of glycosidic bonds. This process converts high-molecular-weight HA into shorter HA chains and oligosaccharide fragments, accompanied by oxidized chain-end structures such as carbonyl- or carboxyl-containing products [9,12,37]. Therefore, Fenton oxidation not only decreases Mn and Mw but also disrupts the physically associated HA network that depends on sufficient chain length, chain overlap, intermolecular interactions, and hydration.

From a polymer-solution perspective, the oxidation-induced deterioration of the HA material can be understood as a transition from a highly overlapped and mutually crowded HA system to a fragmented and weakly interacting polymer system. High-molecular-weight HA does not contribute to viscosity merely through its mass concentration; its semi-flexible chains occupy large hydrodynamic domains, and both hydrodynamic volume and intrinsic viscosity are strongly dependent on molecular weight [30]. Therefore, at a fixed HA concentration, radical-induced chain scission sharply decreases the hydrodynamic volume of individual HA chains, lowers intrinsic viscosity, and reduces the c[η]-dependent chain overlap and mutual macromolecular crowding. The severe decrease in Mn and Mw after Fenton oxidation should therefore be interpreted not only as the formation of shorter chemical fragments but also as the collapse of the physical basis that supports the HA gel-like network.

This mechanism also explains the rheological behavior of the oxidized samples. In concentrated or semi-dilute HA systems, large HA chains require a relatively long relaxation time to find sufficient space and return to their undisturbed conformations. Under shear or oscillatory deformation, this delayed molecular relaxation contributes to shear-thinning behavior and elastic response. After oxidative cleavage, the shortened HA chains possess smaller excluded-volume domains and can move past one another more readily, leading to faster relaxation, lower resistance to deformation, reduced storage modulus, and a low-viscosity, more fluid-like profile. Conversely, the concentration-dependent recovery of storage modulus, apparent viscosity, and shear-thinning tendency after EGT addition indicates that EGT preserved sufficient HA chain length and hydrodynamic overlap to maintain a partially crowded HA network under oxidative stress.

The same chain-scale mechanism can be extended to the flowability, contact-angle, water-retention, and lyophilized-morphology results. When Fenton oxidation reduces HA molecular weight and weakens mutual macromolecular crowding, the system loses resistance to gravitational spreading, resulting in a larger spread area and a lower apparent contact angle on the glass surface. During freezing and lyophilization, relatively intact high-molecular-weight HA chains and their continuous hydrated domains are more capable of supporting a porous block-like matrix after ice sublimation, whereas extensively degraded HA fragments cannot effectively maintain the original gel framework, leading to loose or collapsed morphology [38,39]. Water retention is also reduced because chain shortening disrupts the continuity of hydrophilic polymer domains and decreases the capacity of the HA network to retain bound and physically entrapped water [30]. Thus, the improved flow resistance, higher contact angle, better water retention, and more intact lyophilized/SEM morphology observed in EGT-containing groups can be attributed to preservation of a spatially continuous hydrated HA network.

EGT most plausibly acts upstream of these material-property changes by reducing oxidative attack before extensive HA backbone cleavage occurs. EGT can scavenge reactive oxygen and nitrogen species and modulate transition-metal-ion-mediated radical generation, thereby lowering the effective radical burden in the Fenton system [17,40]. Previous work has also shown that EGT can decrease radical-induced HA degradation in a concentration-dependent manner [19]. Because HA solution properties depend nonlinearly on molecular weight through hydrodynamic volume, intrinsic viscosity, chain overlap, and mutual macromolecular crowding, even partial inhibition of chain scission can produce a pronounced recovery of viscosity, elasticity, spreading resistance, and water retention. In this sense, the stabilizing role of EGT in the present HA system is not the creation of a new chemically cross-linked HA/EGT gel but the preservation of the original HA molecular weight, physical network continuity, and hydration microenvironment under oxidative stress.

Under oxidative conditions, EGT itself may be converted into oxidized derivatives such as ergothioneine disulfide, ergothioneine sulfonic acid, or hercynine, as reported in previous studies [41,42]. However, the FTIR results in this study did not show clear new characteristic peaks indicating covalent bonding between EGT and HA. Therefore, the interaction between EGT and HA in this system is more likely associated with antioxidant protection and the preservation of the original hydrogen-bonding and hydration microenvironment, rather than the formation of a new covalently cross-linked HA/EGT structure. This distinction is important because the stabilizing effect observed here should be interpreted as protection of the existing physically associated HA network, not as the formation of a new chemically cross-linked hydrogel.

High-molecular-weight HA is generally associated with matrix hydration, viscoelasticity, and anti-inflammatory or tissue-protective functions, whereas low-molecular-weight HA fragments and oligosaccharides may promote pro-inflammatory responses, particularly when generated excessively or persistently [7]. In wound-healing materials, excessive oxidative conversion of high-molecular-weight HA into low-molecular-weight degradation fragments may compromise both formulation stability and the maintenance of a hydrated local microenvironment. This interpretation is also consistent with previous wound-healing studies showing that HA can regulate cell migration and wound closure in a molecular-weight-dependent manner [43].

The cell scratch assay should be interpreted in this context. The culture-medium group lacked a hydrated HA matrix, and the EGT solution group provided EGT alone without a polymeric gel-like network. In contrast, the HA and HA/EGT hydrogel groups provided a hydrated polymeric matrix, which may better mimic an extracellular-matrix-like environment that supports fibroblast migration [44]. The finding that EGT solution alone did not show a comparable pro-migratory effect, whereas EGT addition did not weaken HA-associated scratch closure, indicates that HA was the main functional component promoting L929 cell migration. This further supports that the primary role of EGT in this formulation is antioxidant stabilization while preserving HA-associated cell-migration function.

All samples used in the biological experiments and molecular-weight determination were terminally sterilized by autoclaving at 121 °C for 15 min before analysis. We acknowledge that moist-heat sterilization may alter the properties of polymeric materials [45]. However, autoclaving is generally considered to preserve the integrity of biopolymers better than alternative terminal sterilization methods such as γ-irradiation [46]. Importantly, because all formulation groups were subjected to identical sterilization conditions, the differences observed among groups can be attributed to the EGT concentration rather than to the sterilization step. Moreover, because HA-based wound dressings are expected to undergo terminal sterilization before clinical use, stability data obtained after autoclaving reflect material properties under application-relevant conditions, further supporting the translational relevance of the present findings. EGT itself is thermally robust. Its decomposition temperature is 262–265 °C [47], and its concentration remained unchanged after heating at 100 °C for 24 h [24]. Given these results, no substantial degradation of EGT is expected under the autoclaving conditions used (121 °C for 15 min).

This study has several limitations. The biological evaluation was performed using L929 mouse fibroblasts as a reproducible preliminary cell model. Although fibroblasts play important roles in extracellular-matrix deposition and wound repair, models based on primary human dermal fibroblasts, keratinocytes, multicellular co-culture systems, or animal wound-healing models may provide greater biological and translational relevance [44]. Mechanistically, the molecular-weight, rheological, and physicochemical results consistently support a protective effect of EGT against oxidative degradation of HA, but the detailed molecular events underlying this protection were not directly resolved here. Direct analysis of reactive intermediates and EGT oxidation products would provide stronger mechanistic evidence in future work [24]. Sterilization is a further consideration. As described above, the samples were terminally sterilized by autoclaving before the cell experiments. However, because EGT concentration and HA molecular weight were not directly quantified before and after autoclaving, a possible influence of sterilization on the chemical or molecular stability of the formulation cannot be completely excluded.

From a formulation and translational perspective, EGT is a promising candidate for further investigation because of its reported antioxidant activity and its previously demonstrated ability to attenuate oxidative degradation of HA-based materials [19,24]. In addition, synthetic L-ergothioneine has been evaluated by regulatory authorities for specified food uses, with EFSA concluding that it is safe at the proposed use levels assessed [35], providing a useful safety and regulatory background for further development. However, biomedical applications would still require product-specific evaluation of pharmaceutical-grade purity, manufacturing consistency, formulation stability, sterilization compatibility, local tolerance, and route-specific safety. Future studies should therefore focus on optimizing the EGT concentration and formulation composition, incorporating EGT into clinically relevant HA-based wound-dressing or hydrogel platforms, elucidating the molecular mechanism of EGT-mediated antioxidant protection, and validating the findings in more physiologically relevant wound models and animal studies [19,44].

Overall, this study shows that Fenton oxidation affects HA-based wound-healing materials not only by reducing molecular weight but also by transmitting molecular-level chain damage to the physically associated HA network and key material attributes, including rheological behavior, flowability, wettability, water retention, and lyophilized morphology. EGT mitigated these changes without introducing detectable new covalent structures or impairing HA-associated cell migration. These findings support the use of EGT as a candidate antioxidant stabilizing excipient for HA-based wound-healing materials and provide a basis for its further application in other oxidation-sensitive polysaccharide formulations.

## 5. Conclusions

This study evaluated the effect of EGT on the stability of HA-based wound-healing materials and investigated its feasibility as an antioxidant stabilizing excipient. The results showed that EGT exhibited good in vitro biocompatibility toward L929 cells. Multi-stress stability screening indicated that light exposure, high-temperature/high-humidity treatment, and quiescent storage had limited effects on the molecular weight and macroscopic optical properties of the HA system. In contrast, Fenton oxidation markedly induced HA backbone scission. EGT increased the Mn and Mw of HA in a concentration-dependent manner and mitigated oxidation-induced changes in material properties, including reduced storage modulus and apparent viscosity, excessive flowability, altered wettability, and decreased water-retention capacity. The cell scratch assay further demonstrated that HA hydrogel was the main functional component promoting L929 cell migration, whereas EGT addition did not interfere with the pro-migratory effect of HA, and EGT alone did not show an independent migration-promoting effect.

Overall, the primary role of EGT in this HA system can be attributed to antioxidant stabilization. This study provides experimental support for the further development of EGT as a novel antioxidant stabilizing excipient in HA-based wound-healing materials and other oxidation-sensitive formulations.

## Data Availability

The original contributions presented in this study are included in the article/Supplementary Material. Further inquiries can be directed to the corresponding authors.

## Supplementary Materials

The following supporting information can be downloaded at: https://www.mdpi.com/article/10.3390/polym18162033/s1, Table S1: Molecular-weight parameters of HA formulations under different treatment conditions; Figure S1: Representative GPC chromatograms showing the effect of EGT on the molecular-weight distribution of HA under Fenton oxidation.

## Abbreviations

The following abbreviations are used in this manuscript:

HA	hyaluronic acid
EGT	ergothioneine
ROS	reactive oxygen species
GSH	glutathione
GSSG	glutathione disulfide
NAC	N-Acetylcysteine
SEM	scanning electron microscopy
FTIR	Fourier Transform Infrared Spectroscopy

## References

[1] Toole B.P. (2004). Hyaluronan: From Extracellular Glue to Pericellular Cue. Nat. Rev. Cancer.

[2] Buckley C., Murphy E.J., Montgomery T.R., Major I. (2022). Hyaluronic Acid: A Review of the Drug Delivery Capabilities of This Naturally Occurring Polysaccharide. Polymers.

[3] Graça M.F.P., Miguel S.P., Cabral C.S.D., Correia I.J. (2020). Hyaluronic Acid-Based Wound Dressings: A Review. Carbohydr. Polym..

[4] Sekar M.P., Suresh S., Zennifer A., Sethuraman S., Sundaramurthi D. (2023). Hyaluronic Acid as Bioink and Hydrogel Scaffolds for Tissue Engineering Applications. ACS Biomater. Sci. Eng..

[5] Jiang D., Liang J., Noble P.W. (2007). Hyaluronan in Tissue Injury and Repair. Annu. Rev. Cell Dev. Biol..

[6] Wang G., Yang F., Zhou W., Xiao N., Luo M., Tang Z. (2023). The Initiation of Oxidative Stress and Therapeutic Strategies in Wound Healing. Biomed. Pharmacother..

[7] Berdiaki A., Neagu M., Spyridaki I., Kuskov A., Perez S., Nikitovic D. (2023). Hyaluronan and Reactive Oxygen Species Signaling—Novel Cues from the Matrix?. Antioxidants.

[8] Duan J., Kasper D.L. (2011). Oxidative Depolymerization of Polysaccharides by Reactive Oxygen/Nitrogen Species. Glycobiology.

[9] Soltés L., Mendichi R., Kogan G., Schiller J., Stankovska M., Arnhold J. (2006). Degradative Action of Reactive Oxygen Species on Hyaluronan. Biomacromolecules.

[10] McNeil J.D., Wiebkin O.W., Betts W.H., Cleland L.G. (1985). Depolymerisation Products of Hyaluronic Acid after Exposure to Oxygen-Derived Free Radicals. Ann. Rheum. Dis..

[11] Yamazaki K., Fukuda K., Matsukawa M., Hara F., Yoshida K., Akagi M., Munakata H., Hamanishi C. (2003). Reactive Oxygen Species Depolymerize Hyaluronan: Involvement of the Hydroxyl Radical. Pathophysiol. Off. J. Int. Soc. Pathophysiol..

[12] Zhao X., Yang B., Li L., Zhang F., Linhardt R.J. (2013). On-Line Separation and Characterization of Hyaluronan Oligosaccharides Derived from Radical Depolymerization. Carbohydr. Polym..

[13] Li D., Qi S., Ma T., Jiang X., Zhou Z., Chi Y., Qi Z., Li H., Tang E., Zheng Y. (2026). Development of an Injectable Supramolecular Hydrogel System for Prolonged and Controlled Insulin Release in Diabetes Management. Int. J. Biol. Macromol..

[14] Cheah I.K., Halliwell B. (2012). Ergothioneine; Antioxidant Potential, Physiological Function and Role in Disease. Biochim. Biophys. Acta.

[15] Ey J., Schömig E., Taubert D. (2007). Dietary Sources and Antioxidant Effects of Ergothioneine. J. Agric. Food Chem..

[16] Gründemann D., Harlfinger S., Golz S., Geerts A., Lazar A., Berkels R., Jung N., Rubbert A., Schömig E. (2005). Discovery of the Ergothioneine Transporter. Proc. Natl. Acad. Sci. USA.

[17] Fu T.-T., Shen L. (2022). Ergothioneine as a Natural Antioxidant against Oxidative Stress-Related Diseases. Front. Pharmacol..

[18] Paul B.D. (2022). Ergothioneine: A Stress Vitamin with Antiaging, Vascular, and Neuroprotective Roles?. Antioxid. Redox Signal..

[19] Valachova K., Svik K., Biro C., Collins M.N., Jurcik R., Ondruska L., Soltes L. (2020). Impact of Ergothioneine, Hercynine, and Histidine on Oxidative Degradation of Hyaluronan and Wound Healing. Polymers.

[20] Yin X., Chen K., Cheng H., Chen X., Feng S., Song Y., Liang L. (2022). Chemical Stability of Ascorbic Acid Integrated into Commercial Products: A Review on Bioactivity and Delivery Technology. Antioxidants.

[21] Monostori P., Wittmann G., Karg E., Túri S. (2009). Determination of Glutathione and Glutathione Disulfide in Biological Samples: An in-Depth Review. J. Chromatogr. B.

[22] Primas N., Lano G., Brun D., Curti C., Sallée M., Sampol-Manos E., Lamy E., Bornet C., Burtey S., Vanelle P. (2023). Stability Study of Parenteral N-Acetylcysteine, and Chemical Inhibition of Its Dimerization. Pharmaceuticals.

[23] Gulcin İ. (2025). Antioxidants: A Comprehensive Review. Arch. Toxicol..

[24] Liu X., Huang Y., Wang J., Zhou S., Wang Y., Cai M., Yu L., Tang Q. (2020). A Study on the Antioxidant Properties and Stability of Ergothioneine from Culinary-Medicinal Mushrooms. Int. J. Med. Mushrooms.

[25] Liu Y., Wang C., Liu R., Zhao M., Ding X., Zhang T., He R., Zhu S., Dong X., Xie J. (2023). Adhesive Ergothioneine Hyaluronate Gel Protects against Radiation Gastroenteritis by Alleviating Apoptosis, Inflammation, and Gut Microbiota Dysbiosis. ACS Appl. Mater. Interfaces.

[26] Tian X., Guo J., Gu C., Wang H., Wang D., Liao Y., Zhu S., Zhao M., Gu Z. (2024). Ergothioneine-Sodium Hyaluronate Dressing: A Promising Approach for Protecting against Radiation-Induced Skin Injury. ACS Appl. Mater. Interfaces.

[27] Gruber-Bzura B., Bubko I., Mielczarek L., Pogorzelska A., Wiktorska K. (2026). Cytotoxicity in Vitro Assay in 3D vs. 2D L929 Cell Cultures-Comparative Analysis of the Response to the Latex Extracts. PLoS ONE.

[28] Ramakrishnan R., Daly A.C. (2026). Revisiting ISO 10993-5 in Vitro Cytotoxicity Standard Tests for Evaluation of Extracellular Matrix-Based Biomaterials. Int. J. Biomater..

[29] Darby I.A., Zakuan N., Billet F., Desmoulière A. (2016). The Myofibroblast, a Key Cell in Normal and Pathological Tissue Repair. Cell. Mol. Life Sci..

[30] Cowman M.K., Schmidt T.A., Raghavan P., Stecco A. (2015). Viscoelastic Properties of Hyaluronan in Physiological Conditions. F1000Research.

[31] Liang C.-C., Park A.Y., Guan J.-L. (2007). In Vitro Scratch Assay: A Convenient and Inexpensive Method for Analysis of Cell Migration in Vitro. Nat. Protoc..

[32] Molefe P.F., Ghasemishahrestani Z., Khumalo N.P., Bayat A. (2026). The in Vitro Wound-Scratch Assay: Applications, Technical Advances, and Limitations in Wound Healing Research. Int. Wound J..

[33] Joorabloo A., Liu T. (2024). Recent Advances in Reactive Oxygen Species Scavenging Nanomaterials for Wound Healing. Exploration.

[34] Pranantyo D., Yeo C.K., Wu Y., Fan C., Xu X., Yip Y.S., Vos M.I.G., Mahadevegowda S.H., Lim P.L.K., Yang L. (2024). Hydrogel Dressings with Intrinsic Antibiofilm and Antioxidative Dual Functionalities Accelerate Infected Diabetic Wound Healing. Nat. Commun..

[35] Turck D., Bresson J.-L., Burlingame B., Dean T., Fairweather-Tait S., Heinonen M., Hirsch-Ernst K.I., Mangelsdorf I., McArdle H.J., EFSA Panel on Dietetic Products, Nutrition and Allergies (NDA) (2017). Statement on the Safety of Synthetic L-Ergothioneine as a Novel Food—Supplementary Dietary Exposure and Safety Assessment for Infants and Young Children, Pregnant and Breastfeeding Women. EFSA J..

[36] Thomas T.A., Francis R.O., Zimring J.C., Kao J.P., Nemkov T., Spitalnik S.L. (2024). The Role of Ergothioneine in Red Blood Cell Biology: A Review and Perspective. Antioxidants.

[37] Hawkins C.L., Davies M.J. (1996). Direct Detection and Identification of Radicals Generated during the Hydroxyl Radical-Induced Degradation of Hyaluronic Acid and Related Materials. Free Radic. Biol. Med..

[38] Annabi N., Nichol J.W., Zhong X., Ji C., Koshy S., Khademhosseini A., Dehghani F. (2010). Controlling the Porosity and Microarchitecture of Hydrogels for Tissue Engineering. Tissue Eng. B Rev..

[39] Foudazi R., Zowada R., Manas-Zloczower I., Feke D.L. (2023). Porous Hydrogels: Present Challenges and Future Opportunities. Langmuir.

[40] Halliwell B., Tang R.M.Y., Cheah I.K. (2023). Diet-Derived Antioxidants: The Special Case of Ergothioneine. Annu. Rev. Food Sci. Technol..

[41] Servillo L., D’Onofrio N., Casale R., Cautela D., Giovane A., Castaldo D., Balestrieri M.L. (2017). Ergothioneine Products Derived by Superoxide Oxidation in Endothelial Cells Exposed to High-Glucose. Free Radic. Biol. Med..

[42] Jenny K.A., Mose G., Haupt D.J., Hondal R.J. (2022). Oxidized Forms of Ergothioneine Are Substrates for Mammalian Thioredoxin Reductase. Antioxidants.

[43] Kawano Y., Patrulea V., Sublet E., Borchard G., Iyoda T., Kageyama R., Morita A., Seino S., Yoshida H., Jordan O. (2021). Wound Healing Promotion by Hyaluronic Acid: Effect of Molecular Weight on Gene Expression and in Vivo Wound Closure. Pharmaceuticals.

[44] Tracy L.E., Minasian R.A., Caterson E.J. (2016). Extracellular Matrix and Dermal Fibroblast Function in the Healing Wound. Adv. Wound Care.

[45] Bakhrushina E.O., Afonina A.M., Mikhel I.B., Demina N.B., Plakhotnaya O.N., Belyatskaya A.V., Krasnyuk I.I. (2024). Role of Sterilization on in Situ Gel-Forming Polymer Stability. Polymers.

[46] Orçati M.Z., de Moraes M.A., Beppu M.M. (2026). Unraveling Sterilization Effects on Konjac Glucomannan: Insights into a Natural Biopolymer for Biomedical Applications. ACS Omega.

[47] Yadan J.-C. (2022). Matching Chemical Properties to Molecular Biological Activities Opens a New Perspective on L-Ergothioneine. FEBS Lett..

