# Supplementary material for: Effect of Ergothioneine on the Stability of Hyaluronic Acid-Based Wound-Healing Materials
Source: Polymers (Basel). 2026 Aug 21;18(16):2033. doi: 10.3390/polym18162033 (PMC13517268; doi:10.3390/polym18162033)
Supplement: Supplementary file 1 [file polymers-18-02033-s001.zip › polymers-4482536-supplementary.pdf]

Supplementary Material

Effect of Ergothioneine on the Stability of Hyaluronic Acid-Based Wound-Healing Materials

Tianyu Ma [1,†], Shuangshuang Qi [1,†], Junkai Liu [1], Dongjiao Li [1], Xia Li [2,3], Shiyue Hu [2], Fuhua Zheng [2], Ruiyan Wang [2], Yang Su [2,3], Yunjiao Chi [1], Xueqi Zhao [1], Zhen Qin [1,*] and Hao Wu [1,*]

[1] School of Pharmaceutical Engineering, Shenyang Pharmaceutical University, Shenyang 110016, China; matianyu001@stu.syphu.edu.cn (T.M.); qishuangshuang@stu.syphu.edu.cn (S.Q.); liujunkai@stu.syphu.edu.cn (J.L.); lidongjiao@stu.syphu.edu.cn (D.L.); chiyunjiao@stu.syphu.edu.cn (Y.C.); zhaoxueqi@stu.syphu.edu.cn (X.Z.)
[2] Bloomage Biotechnology Co., Ltd, Jinan 250101, China; xiali@bloomagebiotech.com (X.L.); husy@bloomagebiotech.com (S.H.); zhengfh@bloomagebiotech.com (F.Z.); wangry@bloomagebiotech.com (R.W.); tenlions1@sina.com (Y.S.)
[3] Key Laboratory of Regenerative Medicine Technology and Material Transformation of Hainan Province, Bloomage Biotechnology (Hainan) Co., Ltd., Haikou 570003, China
* Correspondence: qinzhen@syphu.edu.cn (Z.Q.); haowu@syphu.edu.cn (H.W.)
† These authors contributed equally to this work.

Table S1. Molecular-weight parameters of HA formulations under different treatment conditions. Data are presented as mean ± SD (n = 3).

(A) Fenton oxidation

Prepared EGT concentration (mg/mL)	Mn (g/mol)	Mw (g/mol)	PDI
0	12,140 ± 620	15,917 ± 1,042	1.313 ± 0.104
0.5	118,127 ± 19,708	216,367 ± 15,233	1.856 ± 0.227
1	181,600 ± 31,731	330,000 ± 29,114	1.842 ± 0.230
2	322,667 ± 31,569	552,600 ± 18,431	1.720 ± 0.112
5	373,667 ± 66,429	573,100 ± 38,287	1.553 ± 0.161

(B) Non-oxidized Fenton control

Prepared EGT concentration (mg/mL)	Mn (g/mol)	Mw (g/mol)	PDI
0	647,900 ± 24,033	816,733 ± 16,974	1.261 ± 0.039
0.5	682,833 ± 13,657	835,533 ± 19,954	1.224 ± 0.025
1	697,267 ± 27,376	832,467 ± 24,729	1.194 ± 0.013
2	767,700 ± 33,200	1,051,667 ± 37,233	1.371 ± 0.045
5	728,333 ± 5,397	982,867 ± 5,244	1.350 ± 0.017

(C) Light exposure

Prepared EGT concentration (mg/mL)	Mn (g/mol)	Mw (g/mol)	PDI
0	700,333 ± 9,075	995,867 ± 12,738	1.422 ± 0.026
0.5	644,100 ± 24,857	911,233 ± 5,773	1.416 ± 0.051
1	654,033 ± 34,853	933,300 ± 17,703	1.430 ± 0.077
2	654,033 ± 24,412	973,433 ± 6,904	1.490 ± 0.046
5	700,633 ± 5,408	982,533 ± 4,607	1.402 ± 0.017

(D) High-temperature/high-humidity

Prepared EGT concentration (mg/mL)	Mn (g/mol)	Mw (g/mol)	PDI
0	729,300 ± 27,698	988,433 ± 56,176	1.354 ± 0.026
0.5	758,967 ± 37,959	1,002,067 ± 43,261	1.321 ± 0.021
1	718,133 ± 39,208	972,933 ± 23,414	1.357 ± 0.046
2	688,500 ± 9,379	912,267 ± 12,604	1.325 ± 0.011
5	728,433 ± 13,410	977,367 ± 8,709	1.342 ± 0.015

(E) Quiescent storage

Prepared EGT concentration (mg/mL)	Mn (g/mol)	Mw (g/mol)	PDI
0	706,233 ± 17,356	944,667 ± 35,966	1.337 ± 0.022
0.5	676,867 ± 17,352	944,833 ± 7,564	1.397 ± 0.042
1	697,700 ± 6,115	960,000 ± 15,290	1.376 ± 0.029
2	702,300 ± 22,732	938,167 ± 17,371	1.336 ± 0.026
5	703,900 ± 26,458	966,967 ± 9,952	1.375 ± 0.042

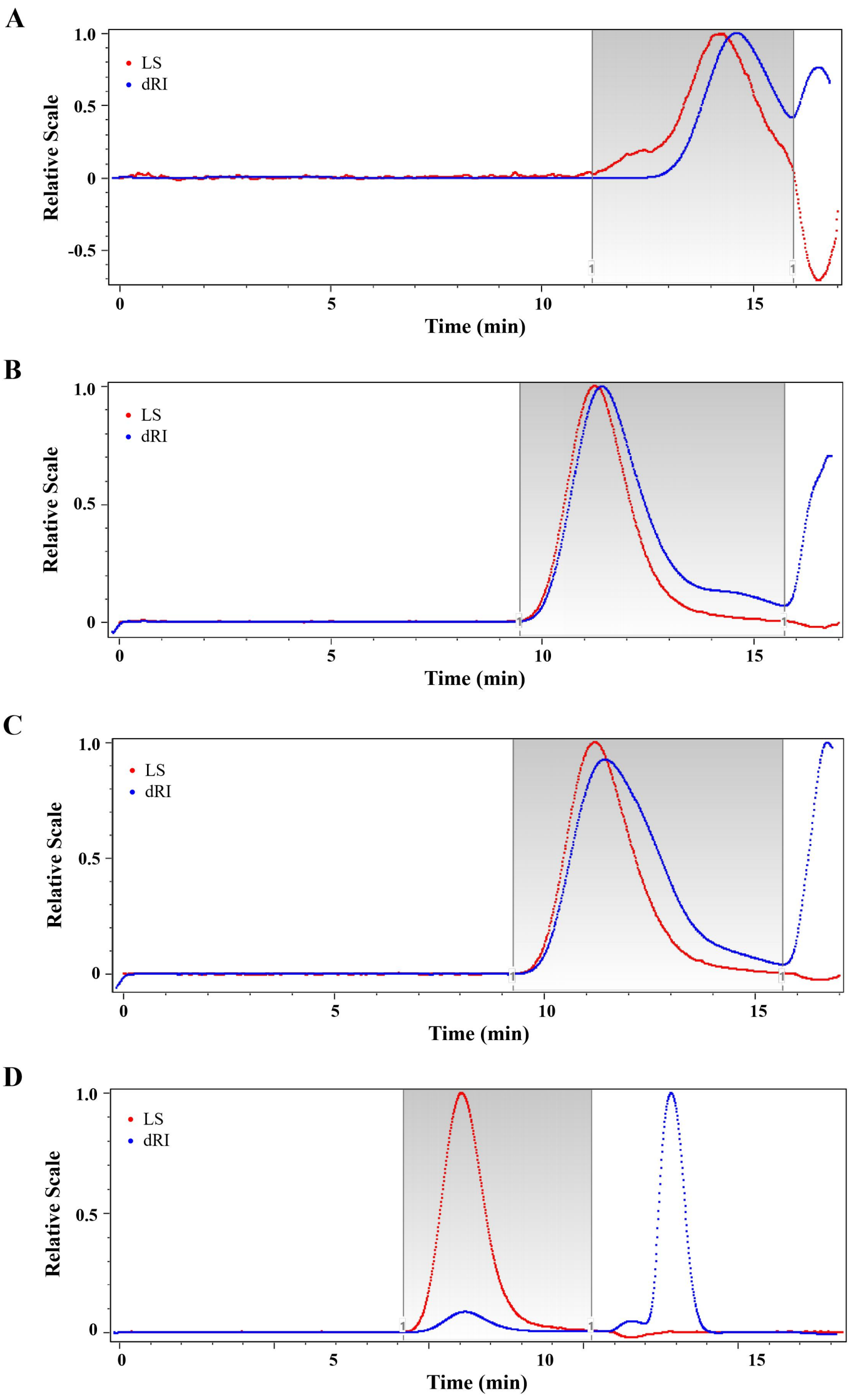

Figure S1. Representative GPC chromatograms showing the effect of EGT on the molecular-weight distribution of HA under Fenton oxidation. (A) Fenton-oxidized HA group. (B) Fenton-oxidized group with EGT prepared at 2 mg/mL. (C) Fenton-oxidized group with EGT prepared at 5 mg/mL. (D) Non-oxidized HA control group. The red line represents the light-scattering (LS) signal, the blue line represents

the differential refractive-index (dRI) signal, and the gray shaded region indicates the integration interval used for molecular-weight calculation.
